# Analyzing DNA Origami Nanostructure Assembly by Dynamic Light Scattering and Nanoparticle Tracking Analysis

**DOI:** 10.1002/smtd.202500295

**Published:** 2025-06-19

**Authors:** Qiaochu Zhang, Xu Chang, Alireza Ebrahimimojarad, Akshay Shah, Fei Zhang, Jinglin Fu

**Affiliations:** ^1^ Center for Computational and Integrative Biology Rutgers University–Camden Camden NJ 08102 USA; ^2^ Department of Chemistry Rutgers University‐Camden Camden NJ 08102 USA; ^3^ Department of Chemistry Rutgers University‐Newark Newark NJ 07102 USA

**Keywords:** DNA origami, dynamic DNA self‐assembly, dynamic light scattering, nanoparticle tracking analysis, size distribution

## Abstract

The field of nucleic acid self‐assembly has advanced significantly, enabling the creation of multi‐dimensional nanostructures with precise sizes and shapes. These nanostructures hold great potential for various applications, including biocatalysis, smart materials, molecular diagnosis, and therapeutics. Here, dynamic light scattering (DLS) and nanoparticle tracking analysis (NTA) are employed to investigate DNA origami nanostructures, focusing on size distribution and particle concentration. Compared to DLS, NTA provided higher resolution in size measurement with a smaller full‐width at half‐maximum (FWHM), making it particularly suitable for characterizing DNA nanostructure. To enhance sensitivity, a fluorescent NTA method is developed by incorporating an intercalation dye to amplify the fluorescence signals of DNA origami. This method is validated by analyzing various DNA origami structures, ranging from 1 and 2D flexible structures to 3D compact shapes, and evaluating structural assembly yields. Additionally, NTA is used to analyze dynamic DNA nanocages that undergo conformational switches among linear, square, and pyramid shapes in response to the addition of trigger strands. Quantitative size distribution data is crucial not only for production quality control but also for providing mechanistic insights into the various applications of DNA nanomaterials.

## Graphical abstract

This figure illustrates the size distribution analysis of reconfigurable DNA nanocages using NTA. It displays three distinct peaks corresponding to particle populations at 43 nm, 73 nm, and 138 nm, each associated with unique nanocage conformations captured by AFMin the insets. These peaks represent the monomer, dimer, and multimer or aggregated DNA nanostructures, respectively. NTA allows the real‐time tracking and high‐resolution measurement of nanoparticle size and concentration, which is useful for characterizing dynamic DNA assemblies in solution.

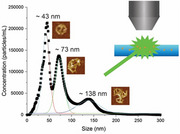

## 1 Introduction

In recent decades, advancements in nucleic acid self‐assembly have enabled the precise design and creation of multi‐dimensional nanostructures with controlled sizes and shapes.^[^ ^1^ ^]^ Due to their programmable and addressable assembly,^[^ ^2^ ^]^ DNA nanostructures are widely used as frameworks to direct the organization of other elements at the nanoscale,^[^ ^3^ ^]^ and to control their spatial arrangement.^[^ ^4^, ^5^ ^]^ Examples include the assembly of multienzymes on DNA nanostructures,^[^ ^6^, ^7^, ^8^ ^]^ the spatial arrangement of ligands,^[^ ^4^, ^9^ ^]^ the engineering of biomimetic nanostructures,^[^ ^10^, ^11^ ^]^ and the spatial regulation of plasmonic nanomaterials.^[^ ^12^, ^13^ ^]^ Functionalized DNA nanostructures hold great potential for various applications such as molecular diagnosis,^[^ ^14^ ^]^ drug and gene delivery,^[^ ^15^, ^16^, ^17^ ^]^ vaccine development^[^ ^18^ ^]^ and energy‐transfer materials.^[^ ^19^, ^20^, ^21^ ^]^ Accurate characterization of the size distribution of DNA nanostructures is essential for production and quality control while providing valuable mechanistic insights into their applications.

Atomic force microscopy (AFM) and transmission electron microscopy (TEM) are widely used to visualize the structural details of 2D and 3D DNA nanostructures.^[^ ^22^, ^23^ ^]^ While these techniques enable super‐resolution imaging of DNA nanostructures fixed on surfaces, they may not accurately reflect the actual sizes and properties of DNA nanostructures in solution. Several experimental studies and simulations have suggested that DNA nanostructures are soft and flexible in solution, often adopting wrapped and twisted conformations.^[^ ^24^, ^25^, ^26^ ^]^ Cryo‐EM provides high‐fidelity information on the structural features of DNA structures.^[^ ^27^, ^28^ ^]^ However, its high cost and time‐consuming nature present significant challenges. As a result, developing a convenient, cost‐efficient, and high‐fidelity method for analyzing the size and shape of DNA nanostructures in solution would be highly beneficial. Such a method would improve the interpretation of size distribution and help identify issues such as aggregation or degradation in solution. Beyond structural characterization, another key challenge is accurately estimating the concentration of DNA nanostructures. The commonly used approach relies on UV absorbance at 260 nm to quantify the total concentration of dsDNA without considering the structural integrity and the purity of DNA nanostructures. Therefore, a method that integrates size distribution data with UV absorbance measurements to assess DNA nanoparticle concentration would provide significant value to the field.

**Figure 1:**
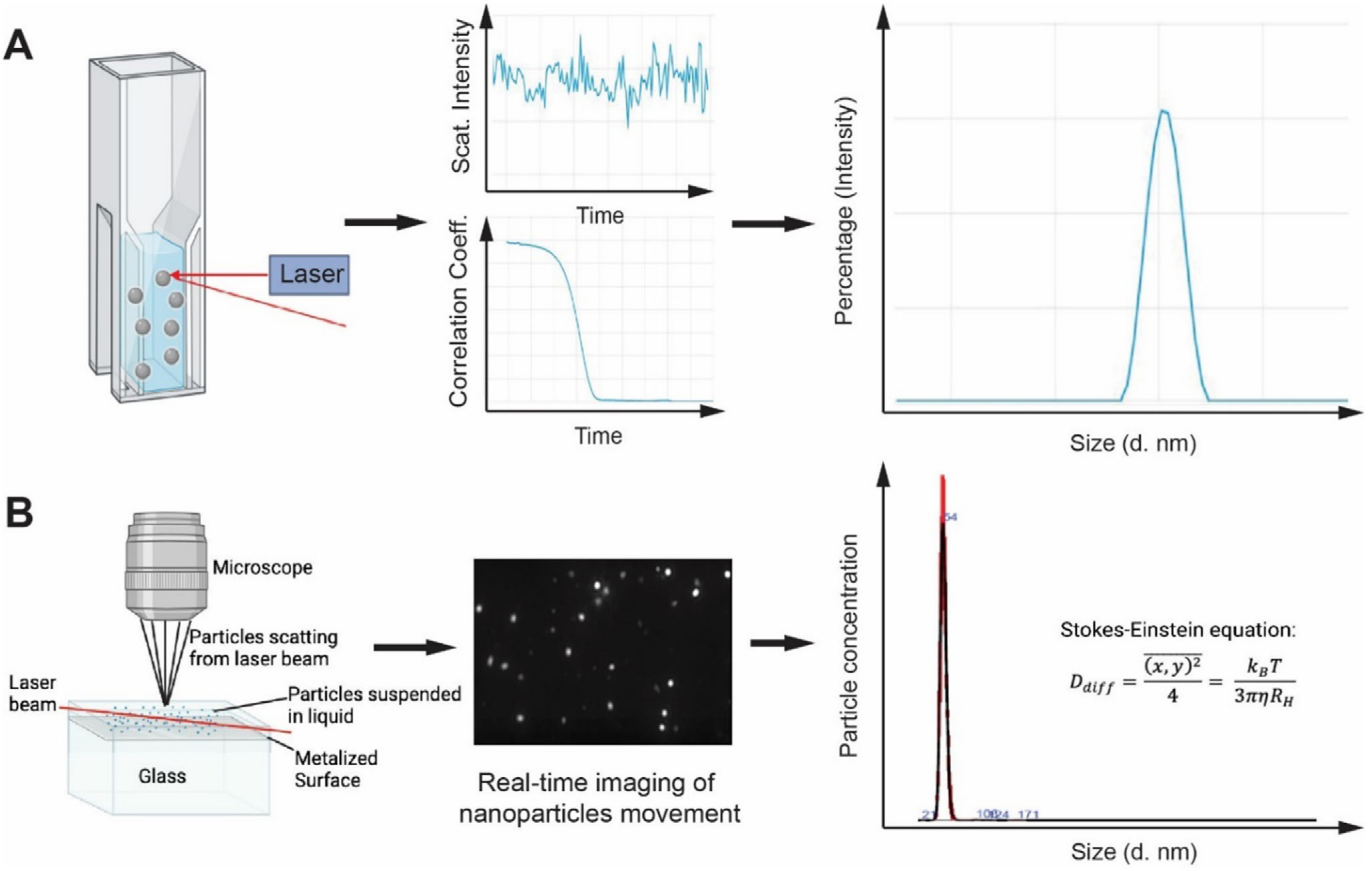
Basic principles of DLS and NTA. A) A hypothetical scenario for the light scattering of nanoparticles and how the correlation function of the scattered light intensity determines particle sizes. DLS used a 637 nm laser and a backscatter at 173 degrees. B) Schematic of the optical configuration used in NTA and the real‐time capture of particle movement by scattering or fluorescence. The size and concentration of nanoparticles individually tracked are analyzed by NTA software based on the Stokes‐Einstein equation. NTA used a 488 nm laser module with the camera perpendicular to the flow channel for imaging the particle movement.

Dynamic Light Scattering (DLS), also known as Quasi‐Elastic Light Scattering, is a non‐invasive, well‐established technique for measuring the size distribution of molecules and nanoparticles, typically in the submicron range, when dispersed in solution.^[^ ^29^, ^30^ ^]^ Brownian motion describes the random movement of particles induced by the solvent molecules' bombardment. Larger particles exhibit slower Brownian motion compared to smaller particles. As shown in **Figure** **1**A, the Brownian motion of particles causes laser light to be scattered at different intensities, fluctuations, and angles. Analyzing these fluctuations in scattering intensity allows for estimating the velocity of Brownian motion. The correlation decay *G(τ)* of the scattered light intensity gives the diffusion coefficient (*D_diff_*) of a particle by:^[^ ^29^, ^31^ ^]^

(1) $$G\left(\tau \right)=A\left[1+B\;\exp\left(-2\Gamma t\;\right)\right]\;$$

where *A* is the baseline of the correlation function, *B* is the intercept of the correlation function. Γ is determined by:

(2) $$\Gamma =D_{diff}\;*q^{2};\;q=\left(\frac{4\pi n}{\lambda_{0}}\right)\;*\sin\left(\frac{\theta }{2}\right)\;$$

where *q* is determined by the refractive index (n) of the solvent, the wavelength of the laser (*λ_0_*), and the scattering angle (*θ*).^[^ ^32^ ^]^ The hydrodynamic radius (*R_H_*) of particles can be estimated by the Stokes‐Einstein Equation (3):

(3) $$R_{H}=\frac{k_{B}T}{3\pi \eta D_{diff}}\;$$

where *k_B_* is Boltzmann's constant, and *η* is the solvent viscosity.

DLS can measure particle sizes ranging from 1 nm to a few µm based on relative number, particle volume, and scattering intensity. Modern DLS systems are adapted to measuring the sizes of biomacromolecules in solution, including proteins, nucleic acids nanostructures, and lipid vesicles. DLS may also be used to estimate the molecular weight of macromolecules and vesicles.^[^ ^33^ ^]^ Light scattering was used to characterize the assembly and disassembly of DNA nanostructures,^[^ ^34^ ^]^ and has been adopted as an analytical tool for DNA nanostructures.^[^ ^35^ ^]^ However, DLS lacks the resolution to analyze multiple components and multimerized nanoparticles.^[^ ^15^ ^]^ Moreover, soft nanoparticles composed of biopolymers exhibit weaker scattering signals than solid and metallic particles,^[^ ^16^ ^]^ making DLS less sensitive for analyzing biomolecular nanoparticles.

Nanoparticle tracking analysis (NTA) is a recently developed technique that enables the direct tracking and analysis of individual nanoparticle diffusion.^[^ ^36^, ^37^ ^]^ In Figure 1B, a laser beam (≈50 µm wide) illuminates a sample chamber, where suspended particles cause light scattering. The scattered light from particles is collected by a long working‐distance objective that is positioned at 90° to the illumination plane.^[^ ^17^, ^19^ ^]^A high‐frame‐rate camera (such as CMOS) operating at ≈30 to a few hundred frames per second is used to record a video of particles moving under Brownian motion within a field of view (e.g., ≈100 µm × 80 µm × 10 µm for Malvern Nanosight). NTA can also detect fluorescence emitted by fluorescent particles to track their movement. The NTA software analyzes the movement traces of individual particles and directly determines the diffusion coefficient by measuring the mean squared displacement of a particle in one, two, or three dimensions. For example, the displacement in two dimensions ($\bar{{\left(x,y\right)}^{2}})$ can be used to calculate the *R_H_* by the Stokes‐Einstein Equation (4):

(4) $$\begin{array}{ccc} D_{diff} & = & \bar{\frac{{\left(x,y\right)}^{2}}{4}}=\frac{k_{B}T}{3\pi \eta R_{H}};\\ & & \;\bar{\frac{{\left(x,y\right)}^{2}}{4}}\;is\;the\;mean\;squared\;displacement\;in\;2D\; \end{array}$$

NTA can measure particle concentration by counting the number of particles detected in a specified volume. Unlike DLS, particle‐by‐particle tracking should provide high‐resolution nanoparticle size distribution and concentration results. Additionally, NTA and DLS complement each other in size measurement. NTA is suitable for larger nanoparticles or aggregates exceeding 50 nm in diameter, while DLS can measure smaller particles as tiny as a few nanometers, such as linear peptides, nucleic acids, and protein monomers. Both DLS and NTA have proven effective in characterizing various nanoscale particles,^[^ ^22^ ^]^ including solid and metallic nanoparticles,^[^ ^22^ ^]^ liposomes,^[^ ^23^ ^]^ extracellular vesicles,^[^ ^24^ ^]^ viruses,^[^ ^25^ ^]^ and protein aggregates.^[^ ^20^ ^]^

Here, we reported a study using DLS and NTA to analyze DNA origami nanostructures for their size distribution and concentrations. We compared the resolution, sensitivity, and reliability of measurements between DLS and NTA. Additionally, we used NTA to analyze the assembly yields of reconfigurable DNA origami structures that can switch between 1D linear, square, and pyramid shapes.

## 2 Results and Discussion

**Figure 2:**
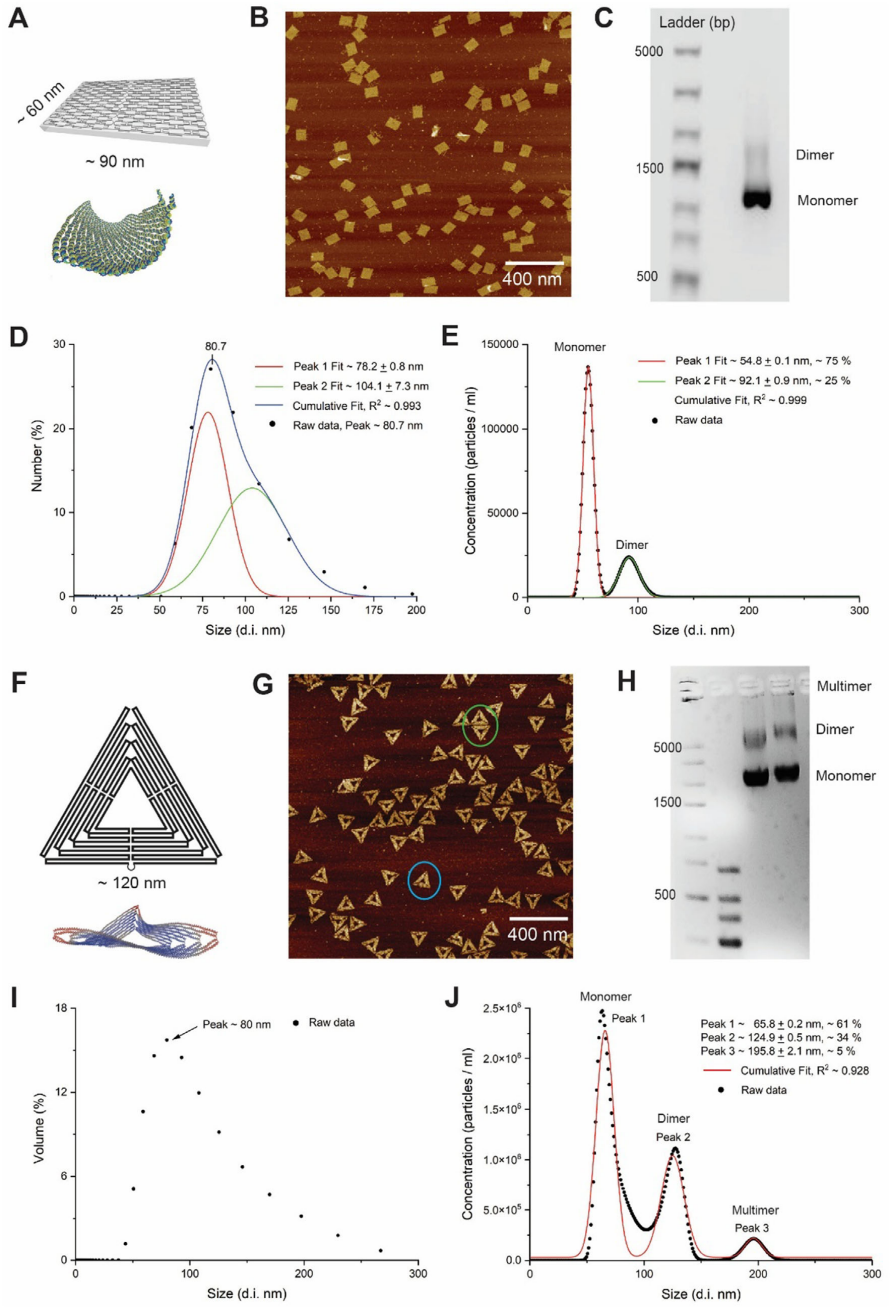
DLS and NTA are used to characterize DNA origami structures by scattering signals. A) A rectangular DNA origami structure with a linear size of ≈60 nm × 90 nm. The simulation indicates a wrapping of the structure in solution. Reproduced with permission.^[^ ^38^ ^]^Copyright 2019, Oxford Academic. The DNA assembly is characterized by B) AFM imaging on a mica surface. Scale bar, 400 nm; and C) agarose gel electrophoresis. D) DLS analysis of rectangular DNA origami at a concentration of ≈7 nm showed a broad peak (depicted by black dots) spanning from ≈50 to 150 nm, with potential distinct peaks at ≈78 and 104 nm as indicated by peak fitting. E) NTA scattering analysis of the same rectangular DNA origami at ≈1 pm showed two distinct peaks at ≈55 nm (primary monomer) and 92 nm (minor dimer). F) A triangular DNA origami structure with a linear size of ≈120 nm. The simulation indicates a wrapping of the structure in solution. Reproduced with permission.^[^ ^39^ ^]^ Copyright 2020, Oxford Academic. The DNA assembly is characterized by G) AFM imaging with examples of monomers (blue circle) and dimers (green circle). Scale bar, 400 nm; and H) agarose gel electrophoresis. I) DLS characterization of triangular DNA origami at a concentration of ≈7 nm exhibited a broad peak ranging from ≈50 to 250 nm, suggesting the multimerization of DNA origamis. J) Fluorescent NTA analysis of the same triangular DNA origami at 1 pm revealed three distinct peaks at ≈66 nm (monomer), 125 nm (dimer), and 196 nm (multimer).

We first characterized a rectangular DNA origami by DLS. Because the particle scattering intensity is not linearly proportional to the size of particles, DLS gives the size distribution based on three modes: number (or population), volume, and signal intensity. In **Figure** **2**A, a rectangular DNA origami was designed with a linear dimension of ≈60 nm × 90 nm. However, the simulation indicated a wrapped structure in a solution smaller than the linear dimension.^[^ ^38^ ^]^ The DNA assembly was first characterized by AFM (Figure 2B), showing a rectangular shape at an average size of ≈60 nm × 100 nm on a 2D mica surface. In Figure 2C, agarose gel electrophoresis verified the assembly of DNA origami and showed a minor band of dimerization. In Figure 2D, the DLS measurement of a rectangular DNA origami (“Number” mode) showed a broad peak spanning from ≈50 to 150 nm, with the highest peak observed at ≈81 nm. The peak fitting analysis identified two potential peaks at ≈78 and 104 nm. In the supporting information Figure S1 (Supporting Information), we discussed the analysis of particle sizes by three modes of “Number”, “Intensity” and “Volume” in DLS. In Figure 2E, the direct track of individual DNA nanoparticles by scattering NTA showed two well‐separated peaks at ≈55 and ≈92 nm. The major population (≈75% by peak area) at ≈55 nm represented a single rectangular DNA origami tile, and the minor population (≈25% by peak area) at the size of ≈92 nm was attributed to the dimer of DNA origami tiles which was also indicated by gel electrophoresis. Interestingly, the size of the DNA origami monomer (≈55 nm d.i.) characterized by DLS is smaller than the designed dimension of ≈60 nm × 90 nm and the AFM characterization. This size discrepancy is mainly attributed to the structural flexibility and wrapping of single‐layer DNA origami in the solution as indicated by the simulation,^[^ ^25^, ^38^ ^]^ electron microscope,^[^ ^26^ ^]^ and X‐ray scattering.^[^ ^24^ ^]^

To further demonstrate the improved resolution of NTA over DLS, we also tested the triangular DNA origami with a linear side length of ≈120 nm, whose structure could also be wrapped or twisted in solution (Figure 2F).^[^ ^39^ ^]^ The triangular DNA origami included more aggregated multimerization than the rectangular DNA origami.^[^ ^40^, ^41^ ^]^ In Figure 2G, AFM showed monomeric triangular shapes and dimeric or multimeric structures on the mica surface. The gel electrophoresis (Figure 2H) also verified significant bands of dimers and multimers for DNA assembly. In Figure 2I, DLS characterization of triangular DNA origami exhibited a broad peak spanning from ≈50 to 250 nm. DNA origami multimers posed a challenge for analysis resolution in DLS. In contrast, NTA revealed three distinct peaks at ≈66 nm (monomer, ≈61% by peak area), 125 nm (dimer, ≈34% by peak area), and 196 nm (multimer, ≈5% by peak area) as shown in Figure 2J. In **Table** **1**, we assessed the resolution of size measurements by the full width at half maximum (FWHM) of peaks for DLS and NTA. NTA analysis consistently exhibited smaller FWHM values for peaks (e.g., 16 nm for peak 1 in rectangular origami) than those obtained with DLS (e.g., 28 nm for peak 1 in rectangular origami), suggesting superior measurement resolution with NTA over DLS. The NTA is also more sensitive than DLS for detecting DNA origami nanostructures. Typically, DLS can detect DNA origami samples at a minimum concentration of a few nanomolars. NTA can detect DNA origami at ≈1 pm, at least a three‐order‐of‐magnitude lower concentration than DLS requires. The design shapes of rectangular and triangular origamis were shown in the Figures S2 and S3 (Supporting Information). We also tested the gel‐purified DNA origamis in DLS, whose size distribution was much narrower, matching the characterization in NTA (Figure S4, Supportng Information).

**Table 1:** Size distribution and resolution of DNA origami as characterized by DLS and NTA.

Origami	Hydrodynamic Diameter (d.i.) by DLS (Number)				Hydrodynamic Diameter (d.i.) by NTA					
	Peak 1		Peak 2		Peak 1		Peak 2		Peak 3	
	Peak center (nm)	FMWH (nm)	Peak center (nm)	FMWH (nm)	Peak center (nm)	FMWH (nm)	Peak center (nm)	FMWH (nm)	Peak center (nm)	FMWH (nm)
Rectangular	78.2 ± 0.8	27.6 ± 2.6	104.1 ± 7.3	48.2 ± 9.2	54.8 ± 0.1	11.1 ± 0.1	92.1 ± 0.9	21.2 ± 0.3	–	–
Triangular	A broad peak with a main size of ≈80 nm, polydispersity ≈0.283				65.8 ± 0.2	18.7 ± 0.5	124.9 ± 0.5	23.7 ± 1.1	195.8 ± 2.1	17.7 ± 5.2

**Figure 3:**
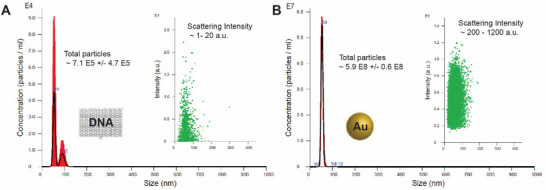
Scattering NTA for characterizing DNA origami and GNPs. A) Scattering NTA detected ≈7.1 × 10^5^ ± 4.7×10^5^ particles/ml for DNA origami, despite the preparation of DNA origami at ≈6.0 × 10^8^ particles/ml (1 pm). The raw scattering intensity for DNA origamis ranged from 1 to 20 a.u. B) Scattering NTA detected 50 nm GNPs at ≈5.9 × 10^8^ ± 6.1 × 10^7^ particles/ml, with a mean size of ≈53.8 ± 0.2 nm. The prepared GNPs were at ≈6.0 × 10^8^ particles/ml. The raw scattering intensity for GNPs ranged from 200 to 1200 a.u.

While scattering NTA demonstrated greater sensitivity and higher resolution than DLS, the relatively weak scattering signals from DNA polymer restricts its ability for more precise measurements, such as accurately determining the concentration of DNA nanoparticles in solution through NTA counting. In **Figure** **3**A, scattering NTA detected ≈7.1 × 10^5^ ± 4.7 × 10^5^ particles/ml for rectangular DNA origami, despite DNA origami being prepared at ≈6.0 × 10^8^ particles/ml (1 pm) as quantified by UV absorbance at 260 nm. The raw scattering intensity for detected DNA origamis ranged from 1–20 a.u. (arbitrary unit), while most DNA origamis were too weak to be detected. The low scattering intensity of DNA origami resulted in poor NTA particle counting, showing a concentration of three orders of magnitude lower than the actual concentration determined by UV absorbance. On the other hand, solid gold nanoparticles (GNPs) exhibited strong scattering signals in solution. In Figure 3B, the scattering NTA measurement of 50 nm GNPs showed a total particle concentration of ≈5.9 × 10^8^ ± 0.6 × 10^8^ particles/ml, which closely matched the prepared nanoparticles concentration of ≈6.0 × 10^8^ particles/ml. The raw scattering intensity of the nanoparticles ranged from 200 to 1200 a.u., making them well‐suited for NTA counting.

**Figure 4:**
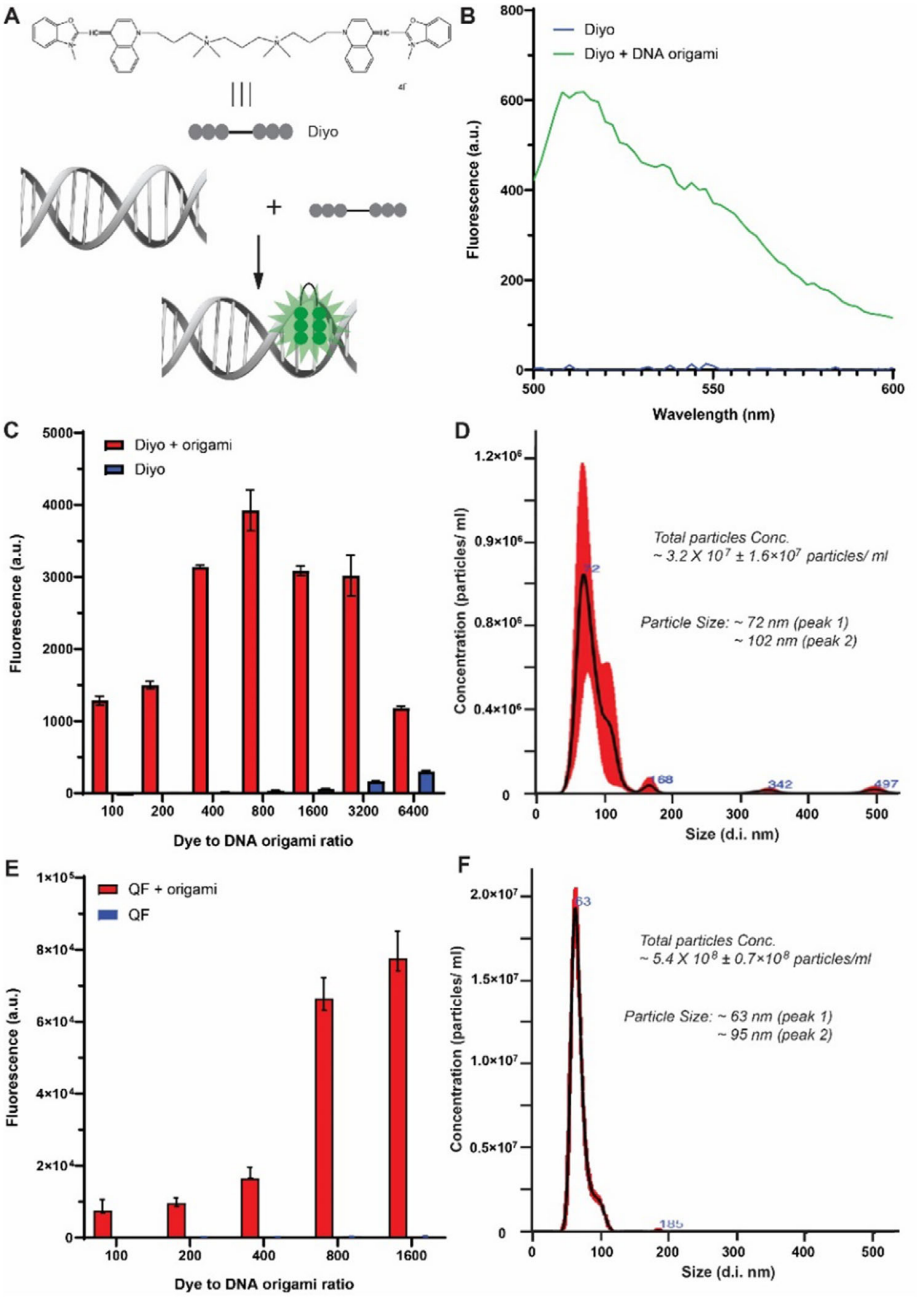
Fluorescent NTA for characterizing the size and concentration of DNA origami. A) Dye molecule (Diyo) is intercalated into double‐stranded DNA to enhance fluorescence. B) Enhanced fluorescence emission by intercalating Diyo into a rectangular DNA origami. C) Titration of the Diyo‐to‐DNA origami ratio for optimizing fluorescence signals. D) Fluorescent NTA of Diyo‐intercalated rectangular DNA origami at a ratio of 800:1. E) Titration of QF‐to‐DNA origami ratio for optimizing fluorescent signals. QF self‐fluorescence is too low to be observed in the graph. F) Fluorescent NTA of QF‐intercalated rectangular DNA origami at a ratio of 800. For all measurements, DNA origami was prepared at ≈6 × 10^8^ particles/ml (≈1 pm). The NTA imaging threshold was set at 2 to collect most nanoparticle signals. Error bar, standard deviation of at least three measurements.

To increase the detection sensitivity, we next used fluorescent NTA to analyze DNA origami nanostructures. To make DNA nanostructures fluorescent, we labeled DNA origami with intercalation dyes. Intercalation dyes generally refer to a group of molecules that can be inserted into planar bases pairing of dsDNA (**Figure** **4**A). The inserted dyes will exhibit much stronger fluorescence than free dye molecules, which are widely used for analyzing dsDNA molecules.^[^ ^42^ ^]^ In Figure 4B, the fluorescence emission of a DiYO‐1 (Diyo) was enhanced by binding to a DNA origami compared to free Diyo. In Figure 4C, by titrating the incubation ratio of Diyo‐to‐DNA origami, the maximal fluorescence emission was obtained when Diyo was added into DNA origami with a molar ratio of 800:1. The higher Diyo‐to‐DNA origami ratio than 800 resulted in a decreased fluorescence signal. In Figure 4D, Diyo‐intercalated, rectangular DNA origami was detected by fluorescent NTA, showing two peaks at ≈72 nm for monomer and ≈102 nm for dimer. The increased particle population at ≈102 nm suggested a potential DNA origami aggregation induced by Diyo intercalation. The total DNA particle concentration was ≈3.2 × 10^7^ ± 1.6 × 10^7^ particles/ml, which was much higher than that given by scattering NTA (≈7.1 × 10^5^ particles/ml). To further enhance the fluorescent signals of DNA origami, we incubated DNA nanostructures with another intercalation dye of QuantiFluor (QF), which was used for quantifying dsDNA. In Figure 4E, we titrated the incubation ratio of QF to DNA origami from 100:1 to 1600:1, and the fluorescence signals increased more than 10 folds for QF‐intercalated DNA origami. Free QF signals were very weak and were ignored in the analysis. Using the optimized incubation ratio of 800, fluorescent NTA characterized QF‐intercalated DNA origami with a primary peak size at ≈63 nm and a minor peak at ≈95 nm. The total concentration of DNA nanoparticles was ≈5.4 × 10^8^ ± 0.7 × 10^8^ particles/ml, which was close to the prepared DNA origami concentration at ≈6.0 × 10^8^ particles/ml. Based on the above results, QF‐intercalated DNA origami was more sensitive than Diyo‐intercalated DNA origami for NTA measurement. To improve the signal‐to‐noise ratio for NTA analysis, the detection threshold of 3 or more is generally used, which results in a lower number of detected particles. In fluorescent NTA, dye‐intercalated, short, and free staple strands typically showed a peak size of less than 30 nm in diameter (Figure S5, Supporting Information), and such a peak from the staple strands can be eliminated by purifying DNA Origami solution.^[^ ^43^, ^44^ ^]^

It is worth noting that both DLS and NTA measurements tend to show relatively smaller hydrodynamic diameters for single‐layer DNA origamis (e.g., rectangular and triangular DNA origami) compared to their designed dimensions and AFM‐based characterization. It is well known that single‐layer DNA origamis are flexible and prone to wrapping in solution, as validated by cryo‐EM,^[^ ^26^ ^]^ SAXS,^[^ ^24^ ^]^ and simulation.^[^ ^25^, ^38^ ^]^ For example, one study reported the floppy 2D DNA nanostructures characterized by cryo‐EM, while AFM only showed extended structures on the mica surface.^[^ ^45^ ^]^ In addition, several publications utilized the wrapping of rectangular DNA origami to form nanotubes for various applications.^[^ ^46^, ^47^ ^]^ Taken together, these findings suggest that the inherent flexibility of single‐layer DNA nanostructures in solution accounts for the smaller hydrodynamic diameters obtained by NTA compared to AFM. A similar observation of the smaller size of single‐layer DNA origami characterization by DLS was also reported in the literature.^[^ ^48^ ^]^

**Figure 5:**
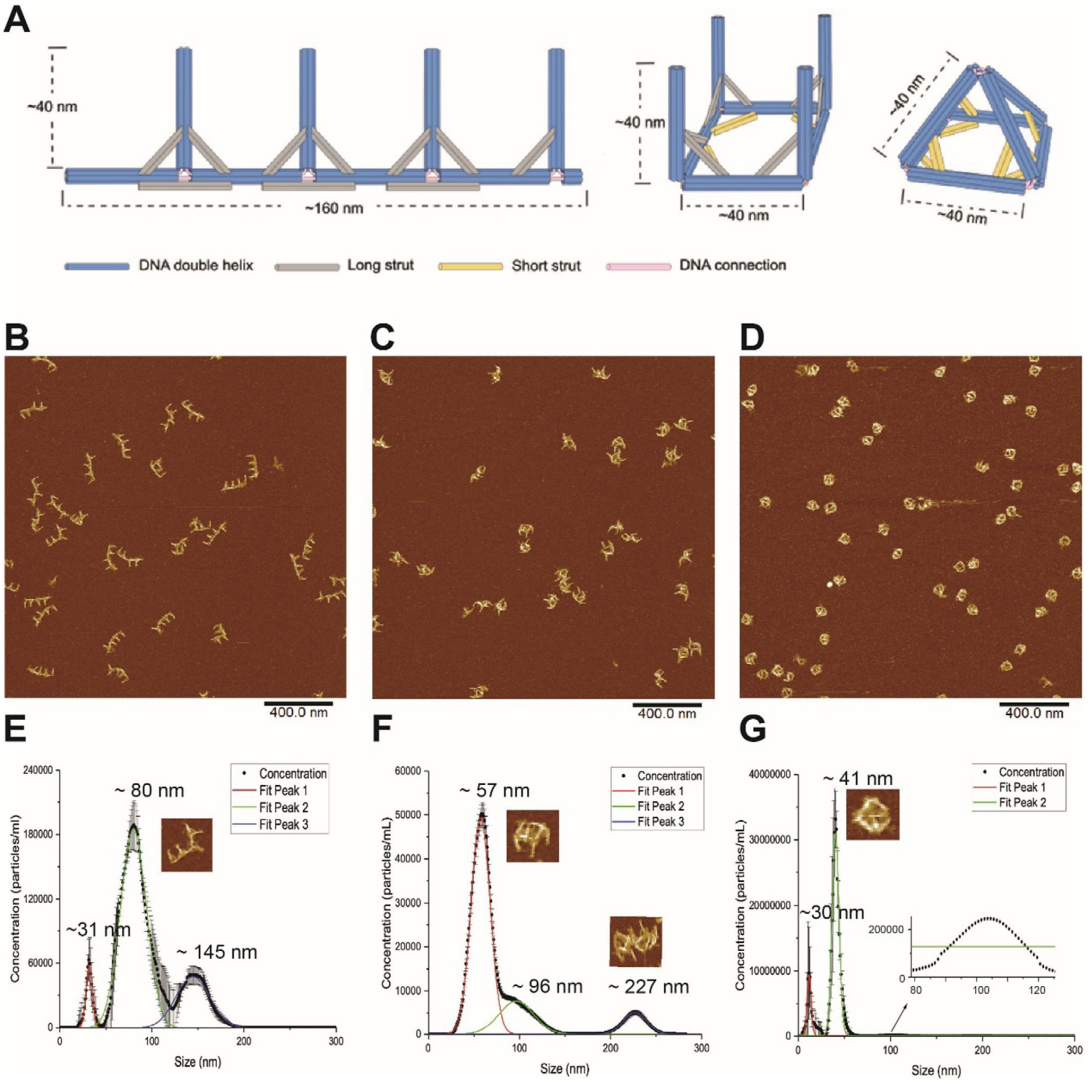
Characterization of multidimensional, DNA nanocage. A) Cartoon model of linear, open square, and pyramid nanocage (from left to right) with size labels. Cylinders of all colors refer to ds DNA. Each dsDNA helix stick (blue) is ≈40 nm. Structures prepared by thermal annealing were characterized by AFM, B) the linear DNA origami, C) the square DNA origami, and D) the pyramid DNA origami nanocages. Scale bar: 400 nm. Fluorescent NTA characterization for E) the linear DNA origami, F) the square DNA origami, and G) the pyramid DNA origami nanocages. ≈1–2 pm DNA origami was used for NTA characterization with an imaging threshold of 3.

With the enhanced sensitivity, we next evaluated using NTA to characterize multidimensional DNA nanocages. A recently published reconfigurable DNA nanocage with multiple conformation^[^ ^49^ ^]^ was used as an example to demonstrate our method. In **Figure** **5**A, we prepared linear shape, open square shape, and pyramid nanocage using thermal annealing. The length of a linear nanocage is ≈160 nm, and the height is ≈40 nm, while each arm of a square‐shaped and pyramid‐shaped nanocage is ≈40 nm. AFM imaging indicated that the yields of direct assembly for the linear nanocage were ≈75.1% ± 0.6% (Figure 5B), the square nanocage ≈84.3% ± 4.9% (Figure 5C), and the pyramid nanocage (Figure 5D) ≈79.3% ± 5.2% as shown in **Table** **2**. The counting of assembly by AFM imaging was detailed in the Figure S6 (Supporting Information). In Figure 5E, fluorescent NTA showed a major peak at ≈80 nm (d.i.) for the linear‐shaped DNA origami and two minor peaks at ≈31 nm (d.i.) and ≈145 nm (d.i.). Linear‐shaped DNA origami showed flexible conformations under AFM that could contribute to the complex peaks observed by NTA. The peak at 80 nm (d.i.) represented the wrapped monomers, which was shorter than the linear length of 160 nm. NTA estimated that the assembly yield for the monomeric linear shape was ≈70.8% by the peak area at 80 nm, similar to AFM counting in Figure 5B. The peak at 145 nm (d.i.) may indicate an extended linear structure or multimer of wrapped structures, whereas the partial structure or free staples contributed to a minor peak at 31 nm or smaller. Combining the two peaks at 80 and 145 nm, the total assembly yield was ≈94.5%. In Figure 5F, the square‐shaped DNA origami showed the size distribution with a major peak at ≈57 nm representing the monomer and a minor shoulder peak at ≈96 nm representing dimers. Another minor peak at a much larger size of ≈227 nm indicated multimerization, which was also observed by AFM and gel (Figure S7, Suppoting Information). The assembly yield of the monomeric square shape was ≈71.2% by the peak area analysis, consistent with the AFM counting yield of ≈84.3%. In Figure 5G, the pyramid‐shaped DNA origami showed a major peak at ≈41 nm, which was well matched with the designed dimension of the pyramid shape. A minor peak at ≈31 nm could be the partial structure or free staples. The assembly yield of the pyramid shape was estimated at ≈84.6% by the peak area analysis of the NTA result, which matched well with the AFM counting yield at 79.3%. When comparing AFM and NTA results for linear, square, and pyramid shapes of DNA origami, it was found that the NTA size measurement was more accurate for the compact and 3D DNA origami objects (e.g., pyramid shape) than these measurements of the flexible 1 and 2D structures (e.g., linear shape). The gel electrophoresis results of the above DNA nanostructures were included in the Figure S7 (Supporting Information).

**Table 2:** AFM and NTA evaluation of the yield of the assembly. NTA uses peak area to calculate the yield. * Although agarose gel electrophoresis can resolve slight band shifts between linear, square, and pyramid structures, the yield estimation by pixel intensity is not sensitive and precise enough for accurate measurement. Thus, the agarose gel electrophoresis is often supplemented by other characterizations, such as AFM, TEM, and NA.

Structure	Yield (%)		
	AFM	NTA	Agarose Gel^*^
Linear	75.1 ± 0.6	70.8 ± 0.5	≈85.8
Square	84.3 ± 4.9	71.1 ± 0.5	≈90.2
Pyramid	79.3 ± 5.2	84.6 ± 0.7	≈88.8
Linear to Square	76.4 ± 2.2	88.8 ± 0.7	≈92.6
Linear to Pyramid	51.6 ± 4.0	43.4 ± 1.1	≈92.9

**Figure 6:**
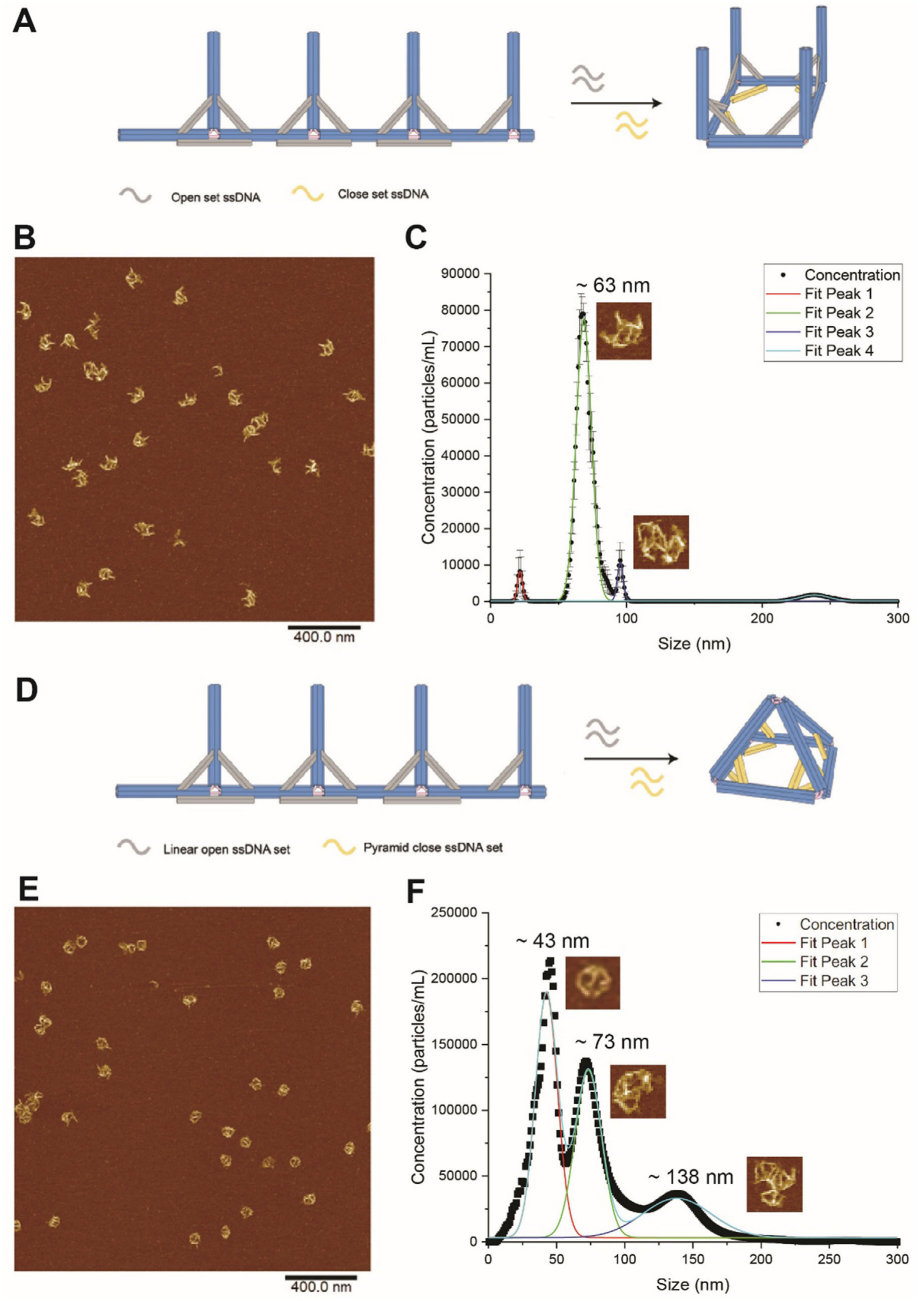
Characterization of the reconfiguration of DNA origami nanocages by AFM and NTA. A) Schematics of reconfiguration process from linear nanocage to square nanocage. Long backbone struts are opened by open‐set DNA and redefined to short backbone struts by close‐set DNA to change the angles of the backbone.B) AFM imaging of switched structures of square DNA origami. C) Fluorescent NTA of the solution for switched structures of square DNA origami. D) Schematics of reconfiguration process from linear nanocage to pyramid nanocage. Long bevel struts are opened by open‐set DNA and redefined to short bevel struts by close‐set DNA to change the angles of arms. E) AFM imaging of switched structures of pyramid DNA origami. F) Fluorescent NTA of the solution for switched structures for pyramid DNA origami. Scale bar: 400 nm. ≈1–2 pm DNA origami was used for NTA characterization with an imaging threshold of 3.

We also used NTA to assess the reconfiguration of the nanocage. Linear shape nanocages were able to reconfigure into open square and pyramid nanocages by replacing staples on DNA struts and redefining the lengths of struts. In **Figure** **6**A, the shape rearranging process from linear to open square nanocage was illustrated in the schematics. AFM imaging indicated a conversion yield from linear to square nanocages at ≈76.4% ± 2.2% (Figure 6B). In Figure 6C, fluorescent NTA detected a peak at ≈63 nm, matching well with the size of the switched square shape. The conversion yield was estimated by peak area at ≈88.8% ± 0.7%. Figure 6D shows the transition from linear to pyramid nanocages using trigger strands. AFM imaging revealed a conversion yield for pyramid nanocages at ≈51.7% ± 4.0% (Figure 6E). In Figure 6F, fluorescent NTA identified a peak at ≈43 nm, matching with the size of the switched pyramid shape, with a yield estimated by peak area at ≈43.4% ± 1.1%. NTA measurements also observed aggregated structures resulting from converting linear shapes to pyramids, such as peaks at 73 and 138 nm. Such aggregations were also observed by AFM. The conversion between the square shape (≈60 nm) and pyramid shape (≈40 nm) was not characterized by NTA due to the similar peak sizes that cannot be resolved clearly.

In Table 2, it was noted that a discrepancy in the assembly yield existed between AFM characterization and NTA counting. AFM and NTA characterize DNA nanostructures using very different mechanisms and conditions. AFM measures DNA nanostructures on the surface of mica, and the structural information for DNA absorbed on the surface may not represent the structural population in the solution. For example, before running AFM, the DNA‐absorbed mica surface will be washed with a buffer or water a few times, which could remove the loosely bound structures on the surface. Therefore, AFM prefers the tightly bound structures that are well‐formed and could overestimate the assembly yield for large DNA origami by ignoring smaller and incomplete structures that are not bound well on the mica surface. In contrast, NTA measures DNA nanoparticles in solution by scattering signals; however, the measurement could be interfered by impurities or salt precipitates from the buffer solution. Thereby, the discrepancy in the yield determination is expected, and we consider the yield difference between NTA (e.g., ≈71.1% for the square shape) and AFM (≈84.3% for the same square shape) acceptable. In most studies, the yield of DNA assembly is often characterized by multiple methods, like AFM, TEM, and gel electrophoresis. The disparity in assembly yields necessitates the development of various techniques to characterize DNA nanostructures in solution and on the surface.

## 3 Conclusion

In summary, we explored using DLS and NTA to analyze DNA origami nanostructures, focusing on size distribution, assembly yield, and particle concentration. Compared to DLS, NTA offered a higher resolution for size measurements with a smaller FWHM, particularly when characterizing the size and population distribution of multiple components. To enhance the sensitivity of NTA measurement, we used intercalation dyes to boost the fluorescence signals of DNA origami. With optimized sensitivity, fluorescent NTA was employed to assess the assembly of a range of DNA origami structures, including 1D linear, 2D rectangular, triangular, and 3D square and pyramid nanocages. We also found NTA size measurements were more accurate for compact 3D DNA origami objects (e.g., pyramid nanocages) than flexible 1D and 2D structures. As shown in Figure 5, the linear structure showed a significant discrepancy between the design (≈160 nm × 40 nm) and NTA measurement (≈80 nm in d.i.). The size discrepancy was also observed for the 2D rectangular and triangular shapes. In contrast, the compact shape of the 2D pyramid structure showed a consistent size between the design (≈40 nm for the side length) and NTA measurement (≈41 nm d.i.). More size comparisons between measurements and the design were summarized in the Table S6 (Supporting Information). Additionally, NTA enabled the quantification of assembly yields, the analysis of monomers, dimers, and multimers in solution, and the characterization of structural switching in dynamic DNA nanostructures.

These findings demonstrate that NTA provides a valuable alternative to assist the characterization of the structural assembly of DNA nanostructures. Both scattering and fluorescent NTA present robust and sensitive methods for accurately characterizing size distribution, multimerization, and particle concentrations of DNA origami nanostructures in solution. These capabilities are essential for ensuring quality control, optimizing material preparation, and supporting various applications in DNA nanotechnology.

## 4 Experimental Section

### Materials

DNA origami staples were prepared using customer‐synthesized oligonucleotides from Integrated DNA Technologies. M13 single‐stranded DNA was purchased from Bayou Biolabs. 50 nm gold nanoparticles (AuNP) were purchased from Sigma–Aldrich. Intercalating dye of DiYO‐1 (λ_ex_ ≈ 483 nm and λ_em_ ≈501 nm) was purchased from AAT Bioques. QuantiFluor dsDNA (λ_ex_≈ 501 nm and λ_em_ ≈531 nm) was ordered from Promega.

### Preparation of Buffer Solutions

All buffers were prepared using either deionized water or distilled water. 1× TAE −12.5 mm Mg^2+^ (pH 8) buffer solution includes 40 mm Tris, 20 mm acetic acid, 2 mm EDTA, and 12.5 mm magnesium acetate, which is prepared by adding 100 mL of a 10× TAE stock solution into 900 mL of deionized water as described in previously published protocols.^[^ ^43^, ^50^ ^]^

### Preparation of DNA Origami

Rectangular and triangular DNA origami structures were designed by TIAMAT and CADNANO, as reported previously.^[^ ^6^, ^40^ ^]^ 100 µL solution containing 20 nm single‐stranded M13mp18 DNA (M13, 7249 nucleotides) and 5 fold molar excess of staple strands were thermally incubated under an annealing program with the temperature gradually transitioning from 95 to 4 °C as detailed in Table S1 (Supporting Information). Subsequently, excess and free staple strands were removed by centrifugation with 100 kD‐cutoff Amicon filters (500 µL) in 1× TAE ‐12.5 mm Mg^2+^ (pH 8) buffer three times.^[^ ^43^, ^51^ ^]^ The concentration of the purified DNA origami solution was determined by the absorbance at 260 nm, with an assumed extinction coefficient of ≈109119009 M^−1^ cm^−1^ for the M13‐scaffolded DNA origami. The complete staple sequences can be found in the Tables S2 and S3 (Supporting Information). The design details and operation of reconfigurable nanocages were described in our previous publication.^[^ ^49^ ^]^ In brief, the reconfiguration of DNA nanocage was achieved by adding open‐set DNA followed by close‐set DNA for strut relaxing and rebinding. To relax struts, open set DNA was added at a 1 to 5 ratio to scaffold DNA and annealed from 37 °C to 20  °C at 1 °C per 20 min. After the incubation of open‐set DNA, close‐set DNA was added at a 1 to 25 ratio to scaffold DNA and annealed from 37 °C to 20 °C at 1 °C per 20 min to redefine struts. Connection DNA was added at a 1 to 5 ratio with the close‐set DNA during the shape rearrangement. The detailed staple sequences are listed in the Tables S4 and S5 (Supporting Information). DNA origami was characterized and purified by agarose gel electrophoresis as described in the published protocol.^[^ ^43^ ^]^

### DLS and NTA Characterization of DNA Nanostructures

A Zetasizer Pro (Malvern Panalytical) was used for DLS experiments, as reported previously.^[^ ^15^, ^52^ ^]^ All buffer solutions used for DLS experiments were filtered by a 0.2 µm syringe filter. Before measurement, a disposal cuvette was rinsed three times with distilled water. 100 µL of ≈5–10 nm DNA origami solution was added into the cuvette to measure DNA nanoparticles' hydrodynamic diameter (d.i.) by light scattering. All experiments ran at room temperature in 1× TAE ‐12.5 mm Mg^2+^ (pH 8) buffer; each sample's running time was ≈5 min (including routine calibration and three repeats).

A Nanosight 300 (Malvern Panalytical) was used for NTA experiments. Before the measurement, all experiment buffer solutions were filtered by a 0.2 µm syringe filter. A DNA sample was diluted in the buffer to a concentration ranging from 10^7^–10^9^ particles/mL. A laser Module of 488 nm was used for NTA measurement. Before the measurement, a PDMS gasket was attached and sealed to the detection glass, forming a solution chamber. The Laser Module was gently pushed into a track to touch the power connector. The “NanoSight NTA 3.4” software was used to operate the NTA measurement. The system was first flushed with water or buffer until the imaging camera showed a clean background. Then, ≈500 µL to 1 mL sample was loaded into a 1 mL syringe, which was mounted onto a pump to control the flow speed. The flow rate was set at 100 in control software for NTA to capture a video of tracking particle movement. The measurement was repeated at least three times with optimized “Screen Gain” and “Camera Level”. A “Detection Threshold” of 2 and 3 was used for analyzing DNA nanoparticles. All experiments were run at room temperature in 1× TAE −12.5 mm Mg^2+^ (pH 8) buffer. For most samples, the running time was 3 min for each sample (three repeats, each 60 s); for samples with relatively weak fluorescence, the running time could be increased to 9 min for each sample (three repeats, each 180 s).

To optimize the dye‐intercalated DNA nanostructures, DNA origami was prepared and diluted in 1× TAE −12.5 mm Mg^2+^ (pH 8) buffer to a concentration of ≈1–5 nm. The intercalating dyes (e.g., DiYo or QuantiFluor) were added into the solution of DNA origami with dye‐to‐DNA origami ratios from 100 to 6400 as needed. The dye and DNA origami were incubated for ≈20 min at room temperature. Then, the dye‐intercalated DNA origami was diluted 1000‐fold for the fluorescent NTA measurements.

### Atomic Force Microscope (AFM) Imaging and Dynamic Light Scattering (DLS)

DNA nanostructures were imaged in liquid by AFM using the published protocol.^[^ ^15^, ^43^, ^51^ ^]^ 1 µL 5 nm DNA nanocage was mixed with 70 µL TAE/Mg^2+^ buffer and 2 µL 100 mm NiCl_2_ solution. The mixture was deposited onto a freshly cleaved mica surface (Ted Pella, Redding, CA). Samples were left to be stable for 5 min. Then, samples were imaged by the “ScanAsyst in Liquid mode” of a Dimension FastScan AFM from Bruker, using a “ScanAsyst‐Fluid+” tip.

### Data Analysis and Fitting

Nanoparticle track analysis software (Marven), GraphPad Prism, and Origin were used to analyze data and plot curves. Peak analysis used the Gauss Fit method in Origin.

## Conflict of Interest

The authors declare no conflict of interest.

## Author Contributions

Q.Z. and X.C. contributed equally to this work. Q.Z., X.C., F.Z., and J.F. designed experiments, Q.Z., A.E., A.S., and X.C. ran experiments and collected data. The manuscript was drafted by Q.Z. and X.C. and revised by J.F. and F.Z. All authors have approved the final version of the manuscript.

## Supporting information



Supporting Information

## Data Availability

The data that support the findings of this study are available in the supplementary material of this article.
