# Supplementary material for: Analyzing DNA Origami Nanostructure Assembly by Dynamic Light Scattering and Nanoparticle Tracking Analysis
Source: Small Methods. 2025 Jun 19;9(8):2500295. doi: 10.1002/smtd.202500295 (PMC12391627; doi:10.1002/smtd.202500295)
Supplement: Supplementary file 1 — Supporting Information [file SMTD-9-2500295-s001.pdf]

small
methods

Supporting Information

for *Small Methods*, DOI 10.1002/smtd.202500295

Analyzing DNA Origami Nanostructure Assembly by Dynamic Light Scattering and Nanoparticle Tracking Analysis

Qiaochu Zhang, Xu Chang, Alireza Ebrahimimojarad, Akshay Shah, Fei Zhang and Jinglin Fu**

Supporting Information

Analyzing DNA Origami Nanostructure Assembly by Dynamic Light Scattering and Nanoparticle Tracking Analysis

Qiaochu Zhang[1], Xu Chang[3], Alireza Ebrahimimojarad[1], Akshay Shah[2], Fei Zhang*[3] and Jinglin Fu*[1,3]

[1]Center for Computational and Integrative Biology, Rutgers University–Camden, Camden, NJ 08102, USA.

[2]Department of Chemistry, Rutgers University–Camden, Camden, NJ 08102, USA.

[3]Department of Chemistry, Rutgers University–Newark, Newark, NJ 07102, USA.

*Corresponding Author: Jinglin Fu, jinglin.fu@rutgers.edu
Fei Zhang, fei.zhang@rutgers.edu

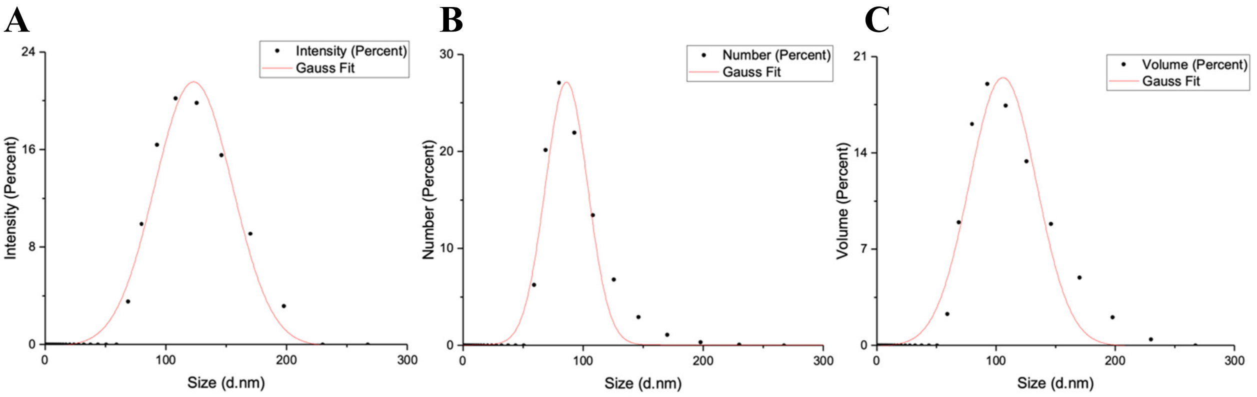

Figure S1. Size distribution tested by DLS. (A) Size distribution based on "Intensity" mode (size: ~ 108 nm). **(B)** Size distribution based on "Number" mode (size: ~ 80 nm). **(C)** Size distribution based on "Volume" mode (size: ~ 93 nm). The "Number" mode prioritized to show nanoparticles with a smaller mean size at ~80 nm because they took up the major population, while the "Intensity" mode preferred to show larger nanoparticles with a mean size at ~ 110 nm due to their strong scattering signals and the "Volume" mode balanced the number and scattering intensity of nanoparticles with a mean size at ~ 93 nm.

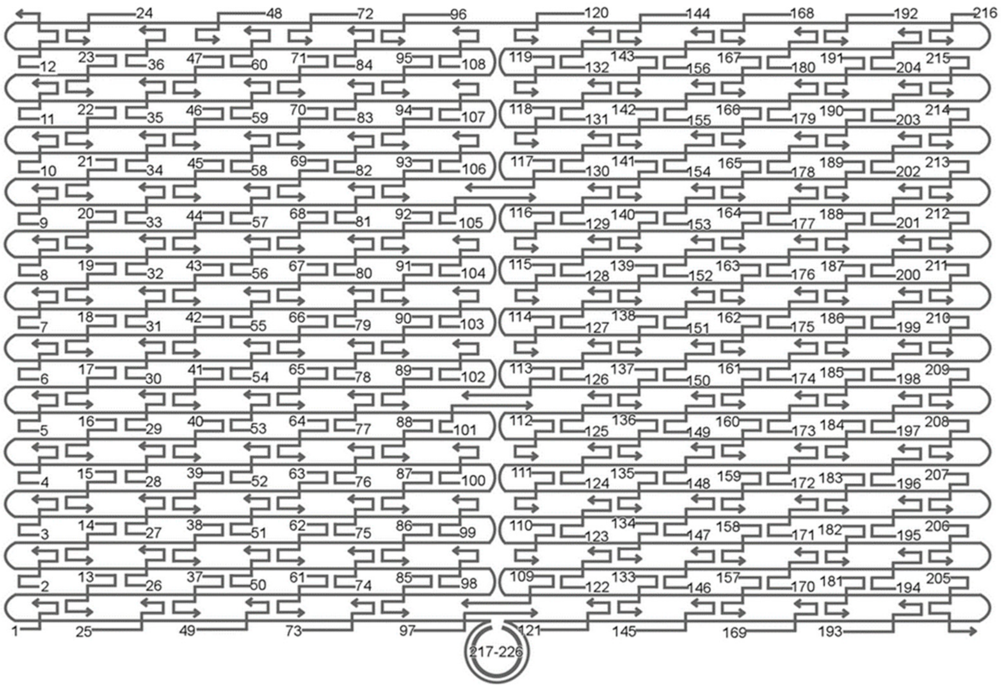

Figure S2. Design of rectangular DNA origami. The size of the origami is ~ 60×100 nm.

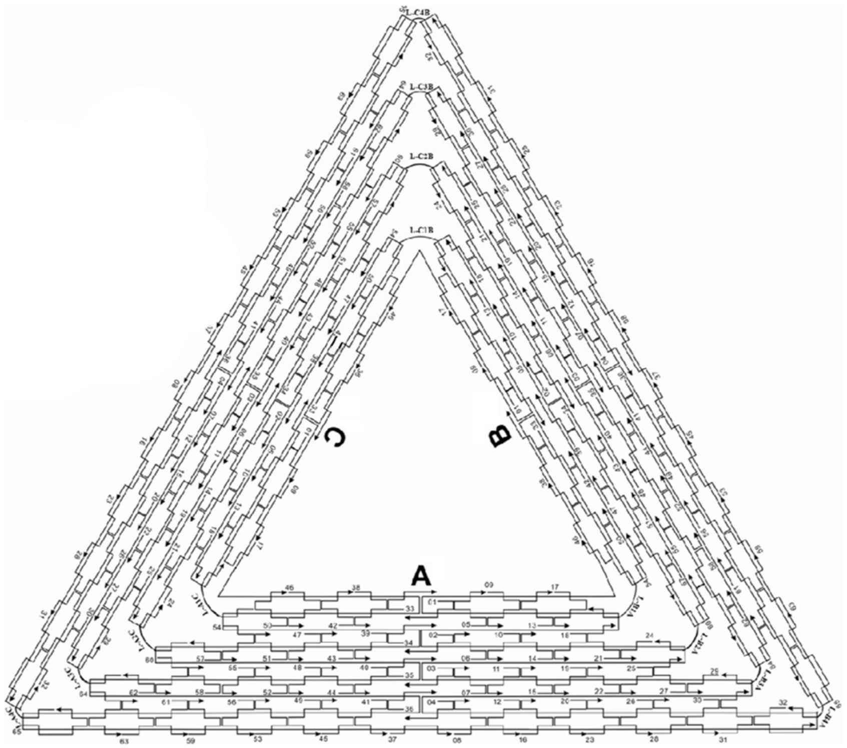

Figure S3. Design of triangular DNA origami. Each side of the origami is ~120 nm.

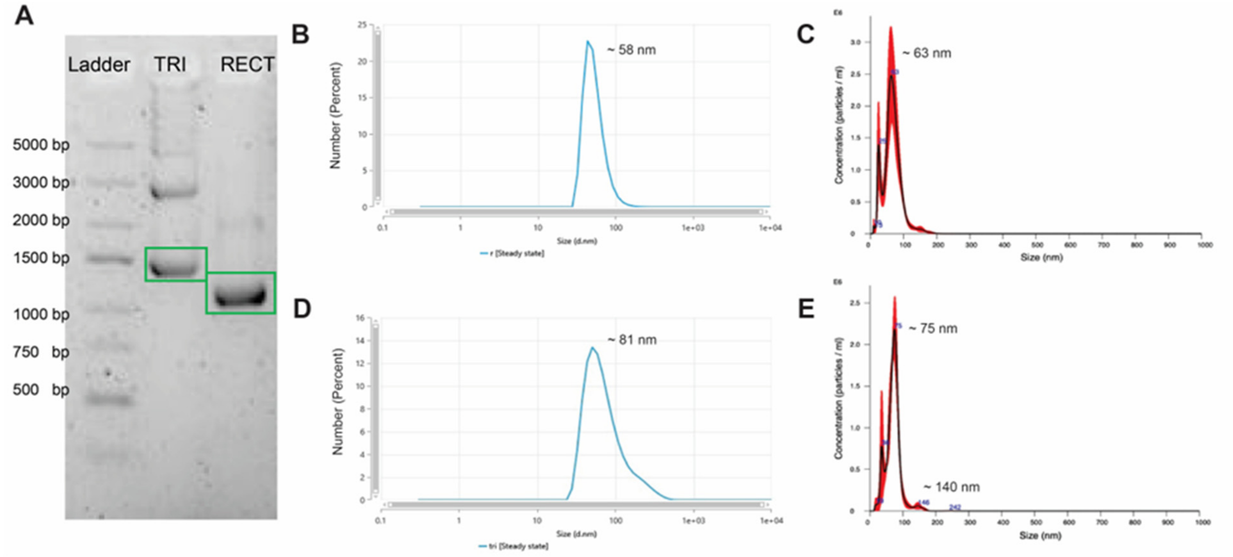

Figure S4. **DLS and NTA characterization of purified DNA origami. (A)** Agarose-gel purification of monomer triangular (TRI) and rectangular (RECT) DNA origamis. A monomer band circled with a green color was cut out from the gel, and DNA origami was extracted from the gel fragments using the published protocol. Purified rectangular DNA origami was characterized by **(B)** DLS and **(C)** scattering NTA. Purified triangular DNA origami was characterized by **(D)** DLS and **(E)** scattering NTA. Minor aggregation exists for gel-purified DNA origami, presumably induced by the spinning process for extracting DNA origami from the gel fragments.

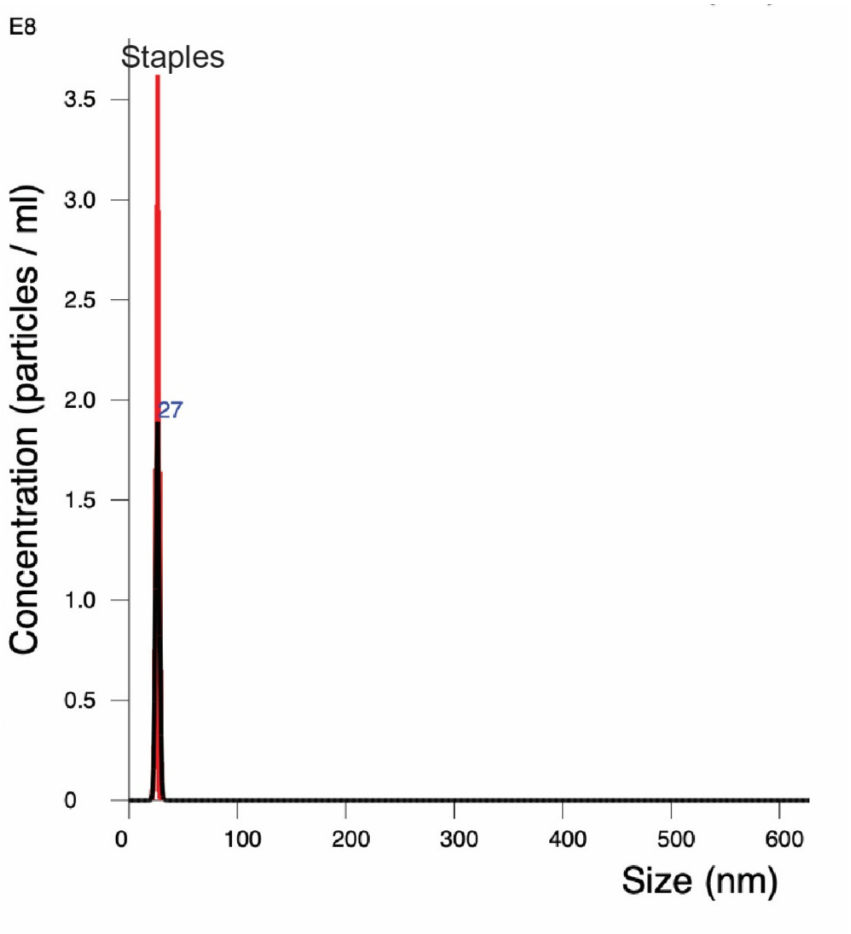

Figure S5. NTA detection of short DNA staples. Size distribution is less than 30 nm (d.i.).

A

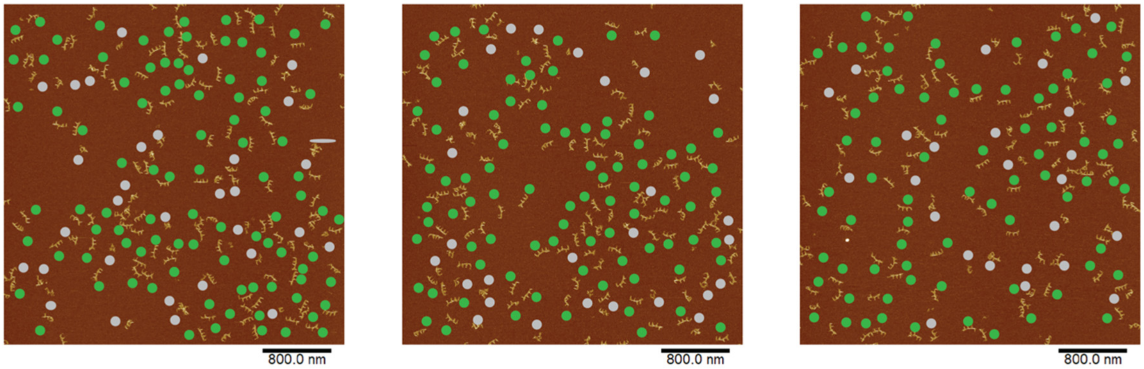

Annealed linear nanocage
Total count: 341
Yield: 75.12 ± 0.548%

B

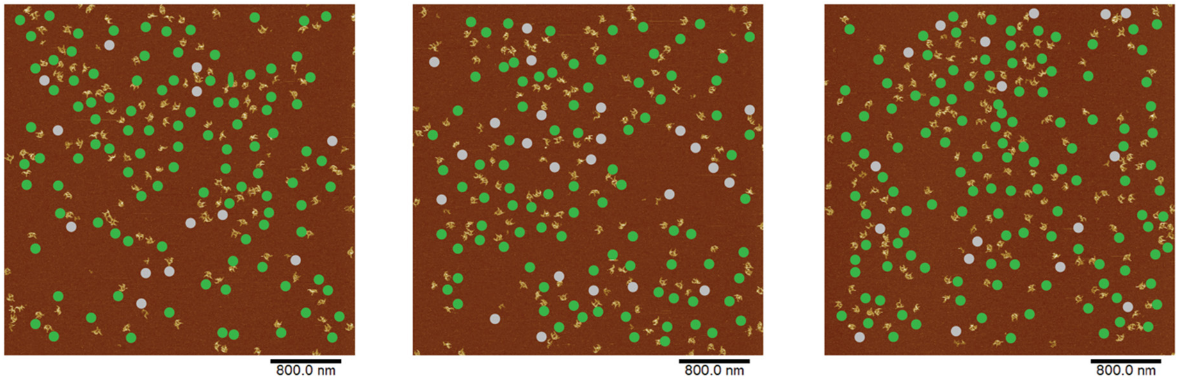

Annealed open square nanocage
Total count: 357
Yield: 84.32 ± 4.85%

C

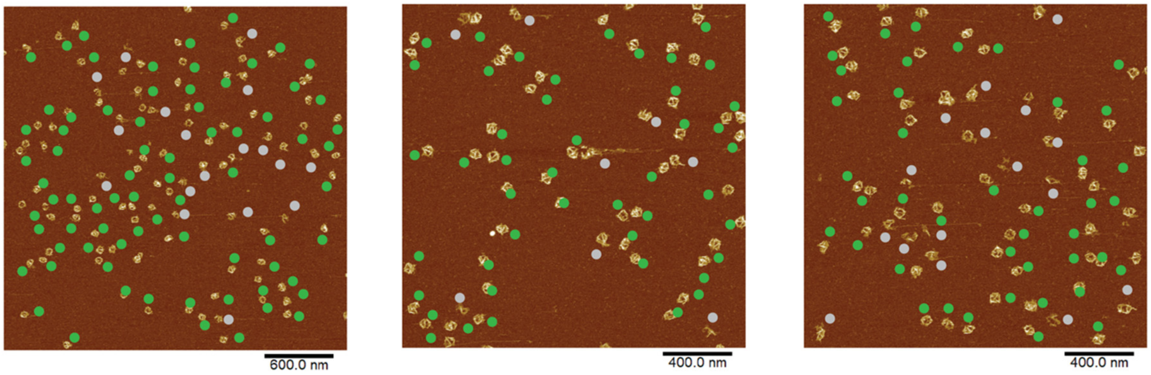

Annealed pyramid nanocage
Total count: 203
Yield: 79.25 ± 5.17%

D

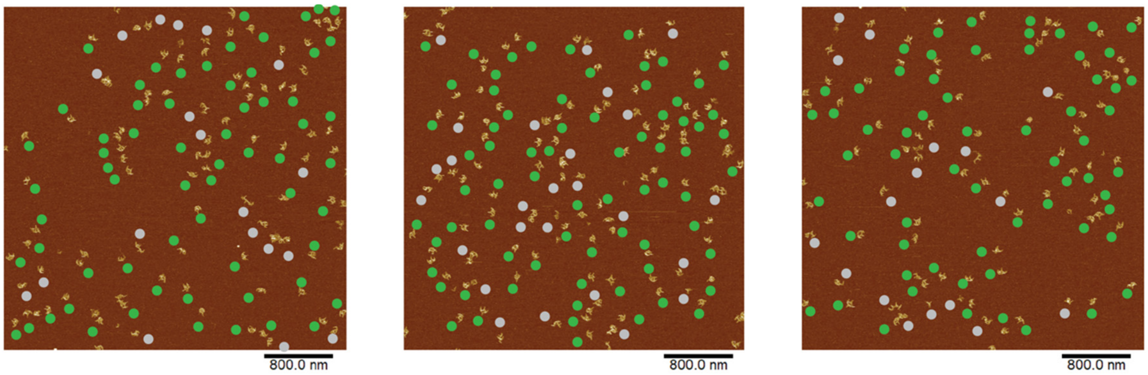

Square nanocage from linear nanocage
Total count: 261
Yield: 76.44 ± 2.21%

Figure S6. AFM images and counting information of annealed nanocages. The green dot refers to qualified linear-shaped nanocages, while the grey dot refers to broken, flawed, and aggregated nanocages. Yield is calculated by dividing the number of green dots by the total number of dots. (A) Annealed open linear nanocage. (B) Annealed open square nanocage. (C) Annealed pyramid nanocage. (D) Square nanocage from linear nanocage.

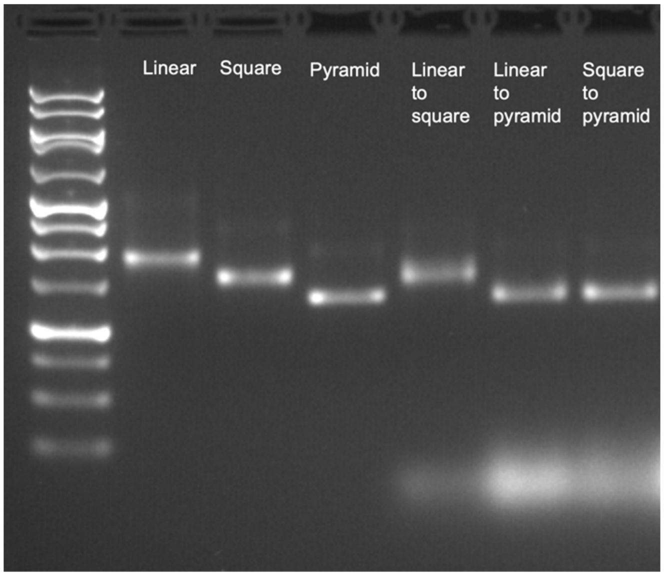

Figure S7. Gel picture of different switchable DNA origami nanocages. Switchable samples include linear, square, pyramid, linear to square, linear to pyramid, and square to pyramid. Aggregation and free staples are also observed in samples.

Table S1. Detailed annealing program for assembling DNA origami tiles.

Temperature (°C)	Gradient
90	30 sec
86-71	1 min/step
70-60	10 min/step
59-30	15 min/step
29-26	10 min/step
25	25 min
4	hold

Table S2. Staple sequences for rectangular DNA origami.

Staple #	Extended staple sequence
13	TGG TTT TTA ACG TCA AAG GGC GAA GAA CCA TC
14	CTT GCA TGC ATT AAT GAA TCG GCC CGC CAG GG
15	TAG ATG GGG GGT AAC GCC AGG GTT GTG CCA AG
16	CAT GTC AAG ATT CTC CGT GGG AAC CGT TGG TG
17	CTG TAA TAT TGC CTG AGA GTC TGG AAA ACT AG
18	TGC AAC TAA GCA ATA AAG CCT CAG TTA TGA CC
19	AAA CAG TTG ATG GCT TAG AGC TTA TTT AAA TA
20	ACG AAC TAG CGT CCA ATA CTG CGG AAT GCT TT
21	CTT TGA AAA GAA CTG GCT CAT TAT TTA ATA AA
22	ACG GCT ACT TAC TTA GCC GGA ACG CTG ACC AA
23	GAG AAT AGC TTT TGC GGG ATC GTC GGG TAG CA
24	ACG TTA GTA AAT GAA TTT TCT GTA AGC GGA GT
25	ACC CAA ATC AAG TTT TTT GGG GTC AAA GAA CG
26	TGG ACT CCC TTT TCA CCA GTG AGA CCT GTC GT
27	GCC AGC TGC CTG CAG GTC GAC TCT GCA AGG CG
28	ATT AAG TTC GCA TCG TAA CCG TGC GAG TAA CA
29	ACC CGT CGT CAT ATG TAC CCC GGT AAA GGC TA
30	TCA GGT CAC TTT TGC GGG AGA AGC AGA ATT AG
31	CAA AAT TAA AGT ACG GTG TCT GGA AGA GGT CA
32	TTT TTG CGC AGA AAA CGA GAA TGA ATG TTT AG
33	ACT GGA TAA CGG AAC AAC ATT ATT ACC TTA TG
34	CGA TTT TAG AGG ACA GAT GAA CGG CGC GAC CT
35	GCT CCA TGA GAG GCT TTG AGG ACT AGG GAG TT
36	AAA GGC CGA AAG GAA CAA CTA AAG CTT TCC AG
37	AGC TGA TTA CAA GAG TCC ACT ATT GAG GTG CC
38	CCC GGG TAC TTT CCA GTC GGG AAA CGG GCA AC
39	GTT TGA GGG AAA GGG GGA TGT GCT AGA GGA TC
40	AGA AAA GCA ACA TTA AAT GTG AGC ATC TGC CA
41	CAA CGC AAT TTT TGA GAG ATC TAC TGA TAA TC
42	TCC ATA TAC ATA CAG GCA AGG CAA CTT TAT TT
43	CAA AAA TCA TTG CTC CTT TTG ATA AGT TTC AT
44	AAA GAT TCA GGG GGT AAT AGT AAA CCA TAA AT
45	CCA GGC GCT TAA TCA TTG TGA ATT ACA GGT AG
46	TTT CAT GAA AAT TGT GTC GAA ATC TGT ACA GA
47	AAT AAT AAG GTC GCT GAG GCT TGC AAA GAC TT
48	CGT AAC GAT CTA AAG TTT TGT CGT GAA TTG CG

49	GTA AAG CAC TAA ATC GGA ACC CTA GTT GTT CC
50	AGT TTG GAG CCC TTC ACC GCC TGG TTG CGC TC
51	ACT GCC CGC CGA GCT CGA ATT CGT TAT TAC GC
52	CAG CTG GCG GAC GAC GAC AGT ATC GTA GCC AG
53	CTT TCA TCC CCA AAA ACA GGA AGA CCG GAG AG
54	GGT AGC TAG GAT AAA AAT TTT TAG TTA ACA TC
55	CAA TAA ATA CAG TTG ATT CCC AAT TTA GAG AG
56	TAC CTT TAA GGT CTT TAC CCT GAC AAA GAA GT
57	TTT GCC AGA TCA GTT GAG ATT TAG TGG TTT AA
58	TTT CAA CTA TAG GCT GGC TGA CCT TGT ATC AT
59	CGC CTG ATG GAA GTT TCC ATT AAA CAT AAC CG
60	ATA TAT TCT TTT TTC ACG TTG AAA ATA GTT AG
61	GAG TTG CAC GAG ATA GGG TTG AGT AAG GGA GC
62	TCA TAG CTA CTC ACA TTA ATT GCG CCC TGA GA
63	GAA GAT CGG TGC GGG CCT CTT CGC AAT CAT GG
64	GCA AAT ATC GCG TCT GGC CTT CCT GGC CTC AG
65	TAT ATT TTA GCT GAT AAA TTA ATG TTG TAT AA
66	CGA GTA GAA CTA ATA GTA GTA GCA AAC CCT CA
67	TCA GAA GCC TCC AAC AGG TCA GGA TCT GCG AA
68	CAT TCA ACG CGA GAG GCT TTT GCA TAT TAT AG
69	AGT AAT CTT AAA TTG GGC TTG AGA GAA TAC CA
70	ATA CGT AAA AGT ACA ACG GAG ATT TCA TCA AG
71	AAA AAA GGA CAA CCA TCG CCC ACG CGG GTA AA
72	TGT AGC ATT CCA CAG ACA GCC CTC ATC TCC AA
73	CCC CGA TTT AGA GCT TGA CGG GGA AAT CAA AA
74	GAA TAG CCG CAA GCG GTC CAC GCT CCT AAT GA
75	GTG AGC TAG TTT CCT GTG TGA AAT TTG GGA AG
76	GGC GAT CGC ACT CCA GCC AGC TTT GCC ATC AA
77	AAA TAA TTT TAA ATT GTA AAC GTT GAT ATT CA
78	ACC GTT CTA AAT GCA ATG CCT GAG AGG TGG CA
79	TCA ATT CTT TTA GTT TGA CCA TTA CCA GAC CG
80	GAA GCA AAA AAG CGG ATT GCA TCA GAT AAA AA
81	CCA AAA TAT AAT GCA GAT ACA TAA ACA CCA GA
82	ACG AGT AGT GAC AAG AAC CGG ATA TAC CAA GC
83	GCG AAA CAT GCC ACT ACG AAG GCA TGC GCC GA
84	CAA TGA CAC TCC AAA AGG AGC CTT ACA ACG CC
85	CCA GCA GGG GCA AAA TCC CTT ATA AAG CCG GC
86	GCT CAC AAT GTA AAG CCT GGG GTG GGT TTG CC
87	GCT TCT GGT CAG GCT GCG CAA CTG TGT TAT CC

88	GTT AAA ATT TTA ACC AAT AGG AAC CCG GCA CC
89	AGG TAA AGA AAT CAC CAT CAA TAT AAT ATT TT
90	TCG CAA ATG GGG CGC GAG CTG AAA TAA TGT GT
91	AAG AGG AAC GAG CTT CAA AGC GAA GAT ACA TT
92	GGA ATT ACT CGT TTA CCA GAC GAC AAA AGA TT
93	CCA AAT CAC TTG CCC TGA CGA GAA CGC CAA AA
94	AAA CGA AAT GAC CCC CAG CGA TTA TTC ATT AC
95	TCG GTT TAG CTT GAT ACC GAT AGT CCA ACC TA
96	TGA GTT TCG TCA CCA GTA CAA ACT TAA TTG TA
97	GAA CGT GGC GAG AAA GGA AGG GAA CAA ACT AT
98	CCG AAA TCC GAA AAT CCT GTT TGA AGC CGG AA
99	GCA TAA AGT TCC ACA CAA CAT ACG AAG CGC CA
100	TTC GCC ATT GCC GGA AAC CAG GCA TTA AAT CA
101	GCT CAT TTT CGC ATT AAA TTT TTG AGC TTA GA
102	AGA CAG TCA TTC AAA AGG GTG AGA AGC TAT AT
103	TTT CAT TTG GTC AAT AAC CTG TTT ATA TCG CG
104	TTT TAA TTG CCC GAA AGA CTT CAA AAC ACT AT
105	CAT AAC CCG AGG CAT AGT AAG AGC TTT TTA AG
106	GAA TAA GGA CGT AAC AAA GCT GCT CTA AAA CA
107	CTC ATC TTG AGG CAA AAG AAT ACA GTG AAT TT
108	CTT AAA CAT CAG CTT GCT TTC GAG CGT AAC AC
109	ACG AAC CAA AAC ATC GCC ATT AAA TGG TGG TT
110	CGA CAA CTA AGT ATT AGA CTT TAC AAT ACC GA
111	CTT TTA CAC AGA TGA ATA TAC AGT AAA CAA TT
112	TTA AGA CGT TGA AAA CAT AGC GAT AAC AGT AC
113	GCG TTA TAG AAA AAG CCT GTT TAG AAG GCC GG
114	ATC GGC TGC GAG CAT GTA GAA ACC TAT CAT AT
115	CCT AAT TTA CGC TAA CGA GCG TCT AAT CAA TA
116	AAA AGT AAT ATC TTA CCG AAG CCC TTC CAG AG
117	TTA TTC ATA GGG AAG GTA AAT ATT CAT TCA GT
118	GAG CCG CCC CAC CAC CGG AAC CGC GAC GGA AA
119	AAT GCC CCG TAA CAG TGC CCG TAT CTC CCT CA
120	CAA GCC CAA TAG GAA CCC ATG TAC AAA CAG TT
121	CGG CCT TGC TGG TAA TAT CCA GAA CGA ACT GA
122	TAG CCC TAC CAG CAG AAG ATA AAA ACA TTT GA
123	GGA TTT AGC GTA TTA AAT CCT TTG TTT TCA GG
124	TTT AAC GTT CGG GAG AAA CAA TAA TTT TCC CT
125	TAG AAT CCC TGA GAA GAG TCA ATA GGA ATC AT
126	AAT TAC TAC AAA TTC TTA CCA GTA ATC CCA TC

127	CTA ATT TAT CTT TCC TTA TCA TTC ATC CTG AA
128	TCT TAC CAG CCA GTT ACA AAA TAA ATG AAA TA
129	GCA ATA GCG CAG ATA GCC GAA CAA TTC AAC CG
130	ATT GAG GGT AAA GGT GAA TTA TCA ATC ACC GG
131	AAC CAG AGA CCC TCA GAA CCG CCA GGG GTC AG
132	TGC CTT GAC TGC CTA TTT CGG AAC AGG GAT AG
133	AGG CGG TCA TTA GTC TTT AAT GCG CAA TAT TA
134	TTA TTA ATG CCG TCA ATA GAT AAT CAG AGG TG
135	CCT GAT TGA AAG AAA TTG CGT AGA CCC GAA CG
136	ATC AAA ATC GTC GCT ATT AAT TAA CGG ATT CG
137	ACG CTC AAA ATA AGA ATA AAC ACC GTG AAT TT
138	GGT ATT AAG AAC AAG AAA AAT AAT TAA AGC CA
139	ATT ATT TAA CCC AGC TAC AAT TTT CAA GAA CG
140	GAA GGA AAA TAA GAG CAA GAA ACA ACA GCC AT
141	GAC TTG AGA GAC AAA AGG GCG ACA AGT TAC CA
142	GCC ACC ACT CTT TTC ATA ATC AAA CCG TCA CC
143	CTG AAA CAG GTA ATA AGT TTT AAC CCC TCA GA
144	CTC AGA GCC ACC ACC CTC ATT TTC CTA TTA TT
145	CCG CCA GCC ATT GCA ACA GGA AAA ATA TTT TT
146	GAA TGG CTA GTA TTA ACA CCG CCT CAA CTA AT
147	AGA TTA GAT TTA AAA GTT TGA GTA CAC GTA AA
148	ACA GAA ATC TTT GAA TAC CAA GTT CCT TGC TT
149	CTG TAA ATC ATA GGT CTG AGA GAC GAT AAA TA
150	AGG CGT TAC AGT AGG GCT TAA TTG ACA ATA GA
151	TAA GTC CTA CCA AGT ACC GCA CTC TTA GTT GC
152	TAT TTT GCT CCC AAT CCA AAT AAG TGA GTT AA
153	GCC CAA TAC CGA GGA AAC GCA ATA GGT TTA CC
154	AGC GCC AAC CAT TTG GGA ATT AGA TTA TTA GC
155	GTT TGC CAC CTC AGA GCC GCC ACC GAT ACA GG
156	AGT GTA CTT GAA AGT ATT AAG AGG CCG CCA CC
157	GCC ACG CTA TAC GTG GCA CAG ACA ACG CTC AT
158	ATT TTG CGT CTT TAG GAG CAC TAA GCA ACA GT
159	GCG CAG AGA TAT CAA AAT TAT TTG ACA TTA TC
160	TAA CCT CCA TAT GTG AGT GAA TAA ACA AAA TC
161	CAT ATT TAG AAA TAC CGA CCG TGT TAC CTT TT
162	CAA GCA AGA CGC GCC TGT TTA TCA AGA ATC GC
163	TTT TGT TTA AGC CTT AAA TCA AGA ATC GAG AA
164	ATA CCC AAG ATA ACC CAC AAG AAT AAA CGA TT
165	AAT CAC CAA ATA GAA AAT TCA TAT ATA ACG GA

166	CAC CAG AGT TCG GTC ATA GCC CCC GCC AGC AA
167	CCT CAA GAA TAC ATG GCT TTT GAT AGA ACC AC
168	CCC TCA GAA CCG CCA CCC TCA GAA CTG AGA CT
169	GGA AAT ACC TAC ATT TTG ACG CTC ACC TGA AA
170	GCG TAA GAG AGA GCC AGC AGC AAA AAG GTT AT
171	CTA AAA TAG AAC AAA GAA ACC ACC AGG GTT AG
172	AAC CTA CCG CGA ATT ATT CAT TTC CAG TAC AT
173	AAA TCA ATG GCT TAG GTT GGG TTA CTA AAT TT
174	AAT GGT TTA CAA CGC CAA CAT GTA GTT CAG CT
175	AAT GCA GAC CGT TTT TAT TTT CAT CTT GCG GG
176	AGG TTT TGA ACG TCA AAA ATG AAA GCG CTA AT
177	ATC AGA GAA AGA ACT GGC ATG ATT TTA TTT TG
178	TCA CAA TCG TAG CAC CAT TAC CAT CGT TTT CA
179	TCG GCA TTC CGC CGC CAG CAT TGA CGT TCC AG
180	TAA GCG TCG AAG GAT TAG GAT TAG TAC CGC CA
181	CTA AAG CAA GAT AGA ACC CTT CTG AAT CGT CT
182	CGG AAT TAT TGA AAG GAA TTG AGG TGA AAA AT
183	GAG CAA AAA CTT CTG AAT AAT GGA AGA AGG AG
184	TAT GTA AAC CTT TTT TAA TGG AAA AAT TAC CT
185	AGA GGC ATA ATT TCA TCT TCT GAC TAT AAC TA
186	TCA TTA CCC GAC AAT AAA CAA CAT ATT TAG GC
187	CTT TAC AGT TAG CGA ACC TCC CGA CGT AGG AA
188	TTA TTA CGG TCA GAG GGT AAT TGA ATA GCA GC
189	CCG GAA ACA CAC CAC GGA ATA AGT AAG ACT CC
190	TGA GGC AGG CGT CAG ACT GTA GCG TAG CAA GG
191	TGC TCA GTC AGT CTC TGA ATT TAC CAG GAG GT
192	TAT CAC CGT ACT CAG GAG GTT TAG CGG GGT TT
193	GAA ATG GAT TAT TTA CAT TGG CAG ACA TTC TG
194	GCC AAC AGT CAC CTT GCT GAA CCT GTT GGC AA
195	ATC AAC AGT CAT CAT ATT CCT GAT TGA TTG TT
196	TGG ATT ATG AAG ATG ATG AAA CAA AAT TTC AT
197	TTG AAT TAT GCT GAT GCA AAT CCA CAA ATA TA
198	TTT TAG TTT TTC GAG CCA GTA ATA AAT TCT GT
199	CCA GAC GAG CGC CCA ATA GCA AGC AAG AAC GC
200	GAG GCG TTA GAG AAT AAC ATA AAA GAA CAC CC
201	TGA ACA AAC AGT ATG TTA GCA AAC TAA AAG AA
202	ACG CAA AGG TCA CCA ATG AAA CCA ATC AAG TT
203	TGC CTT TAG TCA GAC GAT TGG CCT GCC AGA AT
204	GGA AAG CGA CCA GGC GGA TAA GTG AAT AGG TG

217: loop 1	AAC ATC ACT TGC CTG AGT AGA AGA ACT
218: loop 2	TGT AGC AAT ACT TCT TTG ATT AGT AAT
219: loop 3	AGT CTG TCC ATC ACG CAA ATT AAC CGT
220: loop 4	ATA ATC AGT GAG GCC ACC GAG TAA AAG
221: loop 5	ACG CCA GAA TCC TGA GAA GTG TTT TT
222: loop 6	TTA AAG GGA TTT TAG ACA GGA ACG GT
223: loop 7	AGA GCG GGA GCT AAA CAG GAG GCC GA
224: loop 8	TAT AAC GTG CTT TCC TCG TTA GAA TC
225: loop 9	GTA CTA TGG TTG CTT TGA CGA GCA CG
226: loop 10	GCG CTT AAT GCG CCG CTA CAG GGC GC

Table S3. Staple sequences for triangular DNA origami.

Staple #	Extended staple sequence
A01	CGGGGTTTCCTCAAGAGAAGGATTTTGAATTA
A02	AGCGTCATGTCTCTGAATTTACCGACTACCTT
A03	TTCATAATCCCCTTATTAGCGTTTTTCTTACC
A04	ATGGTTTATGTCACAATCAATAGATATTAAAC
A05	TTTGATGATTAAGAGGCTGAGACTTGCTCAGTACCAGGCG
A06	CCGGAACCCAGAATGGAAAGCGCAACATGGCT
A07	AAAGACAACATTTTCGGTCATAGCCAAAATCA
A08	GACGGGAGAATTAACTCGGAATAAGTTTATTTCCAGCGCC
A09	GATAAGTGCCGTCGAGCTGAAACATGAAAGTATACAGGAG
A10	TGTACTGGAAATCCTCATTAAAGCAGAGCCAC
A11	CACCGGAAAGCGCGTTTTCATCGGAAGGGCGA
A12	CATTCAACAAACGCAAAGACACCAGAACACCCTGAACAAA
A13	TTTAACGGTTCGGAACCTATTATTAGGGTTGATATAAGTA
A14	CTCAGAGCATATTCACAAACAAATTAATAAGT
A15	GGAGGGAATTTAGCGTCAGACTGTCCGCCTCC
A16	GTCAGAGGGTAATTGATGGCAACATATAAAAGCGATTGAG
A17	TAGCCCGGAATAGGTGAATGCCCCCTGCCTATGGTCAGTG
A18	CCTTGAGTCAGACGATTGGCCTTGCGCCACCC
A19	TCAGAACCCAGAATCAAGTTTGCCGGTAAATA
A20	TTGACGGAAATACATACATAAAGGGCGCTAATATCAGAGA
A21	CAGAGCCAGGAGGTTGAGGCAGGTAACAGTGCCCG
A22	ATTAAAGGCCGTAATCAGTAGCGAGCCACCCT

A23	GATAACCCACAAGAATGTTAGCAAACGTAGAAAATTATTC
A24	GCCGCCAGCATTGACACCACCCTC
A25	AGAGCCGCACCATCGATAGCAGCATGAATTAT
A26	CACCGTCACCTTATTACGCAGTATTGAGTTAAGCCCAATA
A27	AGCCATTTAAACGTCACCAATGAACACCAGAACCA
A28	ATAAGAGCAAGAAACATGGCATGATTAAGACTCCGACTTG
A29	CCATTAGCAAGGCCGGGGGAATTA
A30	GAGCCAGCGAATACCCAAAAGAACATGAAATAGCAATAGC
A31	TATCTTACCGAAGCCCAAACGCAATAATAACGAAAATCACCAG
A32	CAGAAGGAAACCGAGGTTTTAAGAAAAGTAAGCAGATAGCCG
A33	CCTTTTTTCATTTAACAATTTCATAGGATTAG
A34	TTTAACCTATCATAGGTCTGAGAGTTCCAGTA
A35	AGTATAAAATATGCGTTATACAAAGCCATCTT
A36	CAAGTACCTCATTCCAAGAACGGGAAATTCAT
A37	AGAGAATAACATAAAAACAGGGAAGCGCATTA
A38	AAAACAAAATTAATTAAATGGAAACAGTACATTAGTGAAT
A39	TTATCAAACCGGCTTAGGTTGGGTAAGCCTGT
A40	TTAGTATCGCCAACGCTCAACAGTCGGCTGTC
A41	TTTCCTTAGCACTCATCGAGAACAATAGCAGCCTTTACAG
A42	AGAGTCAAAAATCAATATATGTGATGAAACAAACATCAAG
A43	ACTAGAAATATATAACTATATGTACGCTGAGA
A44	TCAATAATAGGGCTTAATTGAGAATCATAATT
A45	AACGTCAAAAATGAAAAGCAAGCCGTTTTTATGAAACCAA
A46	GAGCAAAAGAAGATGAGTGAATAACCTTGCTTATAGCTTA
A47	GATTAAGAAATGCTGATGCAAATCAGAATAAA
A48	CACCGGAATCGCCATATTTAACAAAATTTACG
A49	AGCATGTATTTCATCGTAGGAATCAAACGATTTTTTGTTT
A50	ACATAGCGCTGTAAATCGTCGCTATTCATTTCAATTACCT
A51	GTTAAATACAATCGCAAGACAAAGCCTTGAAA
A52	CCCATCCTCGCCAACATGTAATTTAATAAGGC
A53	TCCCAATCCAAATAAGATTACCGCGCCCAATAAATAATAT
A54	TCCCTTAGAATAACGCGAGAAAACTTTTACCGACC
A55	GTGTGATAAGGCAGAGGCATTTTCAGTCCTGA
A56	ACAAGAAAGCAAGCAAATCAGATAACAGCCATATTATTTA
A57	GTTTGAAATTCAAATATATTTTAG
A58	AATAGATAGAGCCAGTAATAAGAGATTTAATG
A59	GCCAGTTACAAAATAATAGAAGGCTTATCCGGTTATCAAC
A60	TTCTGACCTAAAATATAAAGTACCGACTGCAGAAC
A61	GCGCCTGTTATTCTAAGAACGCGATTCCAGAGCCTAATTT
A62	TCAGCTAAAAAAGGTAAAGTAATT
A63	ACGCTAACGAGCGTCTGGCGTTTTAGCGAACCCAACATGT

A64	ACGACAATAAATCCCGACTTGCGGGAGATCCTGAATCTTACCA
A65	TGCTATTTTGCACCCAGCTACAATTTTGTTTTGAAGCCTTAAA
B01	TCATATGTGTAATCGTAAAACTAGTCATTTTC
B02	GTGAGAAAATGTGTAGGTAAAGATACAACTTT
B03	GGCATCAAATTTGGGGCGCGAGCTAGTTAAAG
B04	TTCGAGCTAAGACTTCAAATATCGGGAACGAG
B05	ACAGTCAAAGAGAATCGATGAACGACCCCGGTTGATAATC
B06	ATAGTAGTATGCAATGCCTGAGTAGGCCGGAG
B07	AACCAGACGTTTAGCTATATTTTCTTCTACTA
B08	GAATACCACATTCAACTTAAGAGGAAGCCCGATCAAAGCG
B09	AGAAAAGCCCCAAAAAGAGTCTGGAGCAAACAATCACCAT
B10	CAATATGACCCTCATATATTTTAAAGCATTAA
B11	CATCCAATAAATGGTCAATAACCTCGGAAGCA
B12	AACTCCAAGATTGCATCAAAAAGATAATGCAGATACATAA
B13	CGTTCTAGTCAGGTCATTGCCTGACAGGAAGATTGTATAA
B14	CAGGCAAGATAAAAATTTTTAGAATATTCAAC
B15	GATTAGAGATTAGATACATTTCGCAAATCATA
B16	CGCCAAAAGGAATTACAGTCAGAAGCAAAGCGCAGGTCAG
B17	GCAAATATTTAAATTGAGATCTACAAAGGCTACTGATAAA
B18	TTAATGCCTTATTTCAACGCAAGGGCAAAGAA
B19	TTAGCAAATAGATTTAGTTTGACCAGTACCTT
B20	TAATTGCTTTACCCTGACTATTATGAGGCATAGTAAGAGC
B21	ATAAAGCCTTTGCGGGAGAAGCCTGGAGAGGGTAG
B22	TAAGAGGTCAATTCTGCGAACGAGATTAAGCA
B23	AACACTATCATAACCCATCAAAAATCAGGTCTCCTTTTGA
B24	ATGACCCTGTAATACTTCAGAGCA
B25	TAAAGCTATATAACAGTTGATTCCCATTTTTG
B26	CGGATGGCACGAGAATGACCATAATCGTTTACCAGACGAC
B27	TAATTGCTTGGAAGTTTCATTCCAAATCGGTTGTA
B28	GATAAAAACCAAAATATTAAACAGTTCAGAAATTAGAGCT
B29	ACTAAAGTACGGTGTCGAATATAA
B30	TGCTGTAGATCCCCCTCAAATGCTGCGAGAGGCTTTTGCA
B31	AAAGAAGTTTTGCCAGCATAAATATTCATTGACTCAACATGTT
B32	AATACTGCGGAATCGTAGGGGGTAATAGTAAAATGTTTAGACT
B33	AGGGATAGCTCAGAGCCACCACCCCATGTCAA
B34	CAACAGTTTATGGGATTTTGCTAATCAAAAGG
B35	GCCGCTTTGCTGAGGCTTGCAGGGGAAAAGGT
B36	GCGCAGACTCCATGTTACTTAGCCCGTTTTAA
B37	ACAGGTAGAAAGATTCATCAGTTGAGATTTAG
B38	CCTCAGAACCGCCACCCAAGCCCAATAGGAACGTAAATGA
B39	ATTTTCTGTCAGCGGAGTGAGAATACCGATAT

B40	ATTCGGTCTGCGGGATCGTCACCCGAAATCCG
B41	CGACCTGCGGTCAATCATAAGGGAACGGAACAACATTATT
B42	AGACGTTACCATGTACCGTAACACCCCTCAGAACCGCCAC
B43	CACGCATAAGAAAGGAACAACTAAGTCTTTCC
B44	ATTGTGTCTCAGCAGCGAAAGACACCATCGCC
B45	TTAATAAAACGAACTAACCGAACTGACCAACTCCTGATAA
B46	AGGTTTAGTACCGCCATGAGTTTCGTCACCAGGATCTAAA
B47	GTTTTGTCAGGAATTGCGAATAATCCGACAAT
B48	GACAACAAGCATCGGAACGAGGGTGAGATTTG
B49	TATCATCGTTGAAAGAGGACAGATGGAAGAAAAATCTACG
B50	AGCGTAACTACAAACTACAACGCCTATCACCGTACTCAGG
B51	TAGTTGCGAATTTTTTCACGTTGATCATAGTT
B52	GTACAACGAGCAACGGCTACAGAGGATACCGA
B53	ACCAGTCAGGACGTTGGAACGGTGTACAGACCGAAACAAA
B54	ACAGACAGCCCAAATCTCCAAAAAAAAATTTCTTA
B55	AACAGCTTGCTTTGAGGACTAAAGCGATTATA
B56	CCAAGCGCAGGCGCATAGGCTGGCAGAACTGGCTCATTAT
B57	CGAGGTGAGGCTCCAAAAGGAGCC
B58	ACCCCCAGACTTTTTCATGAGGAACTTGCTTT
B59	ACCTTATGCGATTTTATGACCTTCATCAAGAGCATCTTTG
B60	CGGTTTATCAGGTTTCCATTAAACGGGAATACACT
B61	AAAACACTTAATCTTGACAAGAACTTAATCATTGTGAATT
B62	GGCAAAAGTAAAATACGTAATGCC
B63	TGGTTTAATTTCAACTCGGATATTCATTACCCACGAAAGA
B64	ACCAACCTAAAAAATCAACGTAACAAATAAATTGGGCTTGAGA
B65	CCTGACGAGAAACACCAGAACGAGTAGGCTGCTCATTCAGTGA
C01	TCGGGAGATATACAGTAACAGTACAAATAATT
C02	CCTGATTAAAGGAGCGGAATTATCTCGGCCTC
C03	GCAAATCACCTCAATCAATATCTGCAGGTCGA
C04	CGACCAGTACATTGGCAGATTCACCTGATTGC
C05	TGGCAATTTTTAACGTCAGATGAAAACAATAACGGATTCG
C06	AAGGAATTACAAAGAAACCACCAGTCAGATGA
C07	GGACATTCACCTCAAATATCAAACACAGTTGA
C08	TTGACGAGCACGTATACTGAAATGGATTATTTAATAAAAG
C09	CCTGATTGCTTTGAATTGCGTAGATTTTCAGGCATCAATA
C10	TAATCCTGATTATCATTTTGCGGAGAGGAAGG
C11	TTATCTAAAGCATCACCTTGCTGATGGCCAAC
C12	AGAGATAGTTTGACGCTCAATCGTACGTGCTTTCCTCGTT
C13	GATTATACACAGAAATAAAGAAATACCAAGTTACAAAATC
C14	TAGGAGCATAAAAGTTTGAGTAACATTGTTTG
C15	TGACCTGACAAATGAAAAATCTAAAATATCTT

C16	AGAATCAGAGCGGGAGATGGAAATACCTACATAACCCTTC
C17	GCGCAGAGGCGAATTAATTATTTGCACGTAAATTCTGAAT
C18	AATGGAAGCGAACGTTATTAATTTCTAACAAC
C19	TAATAGATCGCTGAGAGCCAGCAGAAGCGTAA
C20	GAATACGTAACAGGAAAAACGCTCCTAAACAGGAGGCCGA
C21	TCAATAGATATTAAATCCTTTGCCGGTTAGAACCT
C22	CAATATTTGCCTGCAACAGTGCCATAGAGCCG
C23	TTAAAGGGATTTTAGATACCGCCAGCCATTGCGGCACAGA
C24	ACAATTCGACAACTCGTAATACAT
C25	TTGAGGATGGTCAGTATTAACACCTTGAATGG
C26	CTATTAGTATATCCAGAACAATATCAGGAACGGTACGCCA
C27	CGCGAACTAAAACAGAGGTGAGGCTTAGAAGTATT
C28	GAATCCTGAGAAGTGTATCGGCCTTGCTGGTACTTTAATG
C29	ACCACCAGCAGAAGATGATAGCCC
C30	TAAAACATTAGAAGAACTCAAACTTTTTATAATCAGTGAG
C31	GCCACCGAGTAAAAGAACATCACTTGCCTGAGCGCCATTAAAA
C32	TCTTTGATTAGTAATAGTCTGTCCATCACGCAAATTAACCGTT
C33	CGCGTCTGATAGGAACGCCATCAACTTTTACA
C34	AGGAAGATGGGGACGACGACAGTAATCATATT
C35	CTCTAGAGCAAGCTTGCATGCCTGGTCAGTTG
C36	CCTTCACCGTGAGACGGGCAACAGCAGTCACA
C37	CGAGAAAGGAAGGGAAGCGTACTATGGTTGCT
C38	GCTCATTTTTTAACCAGCCTTCCTGTAGCCAGGCATCTGC
C39	CAGTTTGACGCACTCCAGCCAGCTAAACGACG
C40	GCCAGTGCGATCCCCGGGTACCGAGTTTTTCT
C41	TTTCACCAGCCTGGCCCTGAGAGAAAGCCGGCGAACGTGG
C43	ACGTTGTATTCCGGCACCGCTTCTGGCGCATC
C44	CCAGGGTGGCTCGAATTCGTAATCCAGTCACG
C45	TAGAGCTTGACGGGGAGTTGCAGCAAGCGGTCATTGGGCG
C46	GTTAAAATTCGCATTAATGTGAGCGAGTAACACACGTTGG
C47	TGTAGATGGGTGCCGGAAACCAGGAACGCCAG
C48	GGTTTTCCATGGTCATAGCTGTTTGAGAGGCG
C49	GTTTGCGTCACGCTGGTTTGCCCCAAGGGAGCCCCCGATT
C50	GGATAGGTACCCGTCGGATTCTCCTAAACGTTAATATTTT
C51	AGTTGGGTCAAAGCGCCATTCGCCCCGTAATG
C52	CGCGCGGGCCTGTGTGAAATTGTTGGCGATTA
C53	CTAAATCGGAACCCTAAGCAGGCGAAAATCCTTCGGCCAA
C54	CGGCGGATTGAATTCAGGCTGCGCAACGGGGGATG
C55	TGCTGCAAATCCGCTCACAATTCCCAGCTGCA
C56	TTAATGAAGTTTGATGGTGGTTCCGAGGTGCCGTAAAGCA
C57	TGGCGAAATGTTGGGAAGGGCGAT

C58	TGTCGTGCACACAACATACGAGCCACGCCAGC
C59	CAAGTTTTTGGGGTCGAAATCGGCAAAATCCGGGAAACC
C60	TCTTCGCTATTGGAAGCATAAAGTGTATGCCCGCT
C61	TTCCAGTCCTTATAAATCAAAAGAGAACCATCACCCAAAT
C62	GCGCTCACAAGCCTGGGGTGCCTA
C63	CGATGGCCCACTACGTATAGCCCGAGATAGGGATTGCGTT
C64	AACTCACATTATTGAGTGTTGTTCCAGAAACCGTCTATCAGGG
C65	ACGTGGACTCCAACGTCAAAGGGCGAATTTGGAACAAGAGTCC
Link-A1C	TTAATTAATTTTTACCATATCAAA
Link-A2C	TTAATTTCATCTTAGACTTTACAA
Link-A3C	CTGTCCAGACGTATACCGAACGA
Link-A4C	TCAAGATTAGTGTAGCAATACT
Link-B1A	TGTAGCATTCCTTTTATAAACAGTT
Link-B2A	TTTAATTGTATTTCCACCAGAGCC
Link-B3A	ACTACGAAGGCTTAGCACCATTA
Link-B4A	ATAAGGCTTGCAACAAAGTTAC
Link-C1B	GTGGGAACAAATTTCTATTTTTGAG
Link-C2B	CGGTGCGGGCCTTCCAAAAACATT
Link-C3B	ATGAGTGAGCTTTTAAATATGCA
Link-C4B	ACTATTAAAGAGGATAGCGTCC
Loop	GCGCTTAATGCGCCGCTACAGGGC

Table S4. DNA staples for linear branch shape DNA nanocage.

Name	Sequence
Body staples	
1	CGGACAGCTTTATAAATATTCATAATGTTTAGAGGAT
2	TTTTTAACCGTTCTGTGAGCGTCAAATATCTGCGGAA
3	CTATCATACAATTAGAGAACAGTTGATTCCCGCAACTACATT
4	AGAGGTCTGGATTTGCCAGATTCATTCC
5	AGTCACAGGTCACTGGATAGCGTCCAATACGCGGAAGCAAA
6	CATCAATTCAAGAGGTAAGTACGGTGT
7	TAGCATTGGCCTTTAAGGGTAATAGTAAAAAAGAAGTGCAA
8	GCTGCGCAACTGTTGTTGACGACG
9	AAAGCGCCATCGCTATTCCAGG
10	ACGTTAATATTTTGTGCGTAGAT
11	TTTTTGATAGGTCACGTTGGTGGATTGACCGTAATGGTTTTT
12	GGGCGCATTATCCGCATAAAGTGTTTTTTCTT
13	ATCTGCCAGTCTGTTTCCCAGTGAGAC
14	ACAGTATAATTCGTGATTGCCCTGACGTTGT
15	GTTTTCCCAGTCACTCGGTGCGGGCC
16	GCAAGCGGTGGGTGGAATAAATTTTTGTAGCTTAAATTGTGC
17	GGCCCTGAACAGCTAATCATGGTC
18	TCTTTCGCCATAGGAAGATCGCACTGATCCCCCGAC
19	AGCTGGCGAGGAAACCGCTTTCCGGCACCGCTTTTTT
20	GCATAGCCTGGGGTGCCTCGGAAGCTCACAATTCCACACAATTTTT
21	TTTTTCTGCAGGTCGACTCTAGAGCCAGCCAAGGC
22	ATAGTTGAGGGGTATAAGCAAATATCCCAAAATGCA
23	AAAAGGGTACCGAGCTCGCGGCCTCTCAG
24	GGCCAGTGCCGGTAACGACGCC
25	TTTTTCATACGAGCAATGAGTGAACAAACGTAAAATTC
26	TTCACTGTGTGAAATTGTCGTAACCGTAA
27	GGGCAGAGAGTACAGGAAGAGGAAGGGCGATCACCGCCT
28	TAAACGCCAGACTGACCAACTTTTCGGAACGACATTGTGAAT
29	TTTTTTGCCGTAAATTTTATACCTGACGAGAAGGGAAC
30	AGGGCTTATAACATTCAACTAATTCAGGTAGATACC
31	CCCACTTTAATGGCGCAGACGGTCAATCATAATGGTT
32	TTTTTCTATTAAAGAACGTGGACTTTGGGAAAACAACATT
33	GGGCGAAAAACTCATTAAAGATTCATCAG
34	GGCGATGGCCTTATGCGCATGTTACTT
35	TCGTCCATCAACATTAAATAGCTGATCATCAATATGATATTCTTTTT

36	ATATGTCATTGCCTGAGAATCTACAAGTAG
37	CGAAATCCTGAGAAGTGTGCACTAATTTTTTGGGGTCGAGGTTTTT
38	GGATTGCATCCTAACGGGAAAAATCTACGTTAATTTTT
39	AAAGAGGGAAAGCCGGCGCGACCTGCTTAGGAATACCAATCA
40	TACCCACTACGCCCGATTTAGAGCTTGACGATTCAG
41	TAATTTCAAAATCAAGATCGGAACCC
42	TTTTTTAAATTGGGCTTGAGAACACCAGAACGAGTAGTTTTT
43	AGTCAGGACGCCAACGTTTGTTCCAGTTTGGTCCAGTCAAGC
44	AGCCGAAATCCGAACGTGGCGAGTAGGGAGCCTGAACCATCA
45	TTTTTTAAAACGAAAAAAAGACTCACTGCCCGCTTAACAAGAGTCCATTTTT
46	ATTACAGAAGCAGGGAAACCTGTCCCTTGAGTGCAAA
47	TTGAGATTCATTTTAAGAACTGGCCGTCTAAGCCCGAGAT
48	CGCCTGATAAATTTTTTTTTGTGGAAAGAGG
49	TATAGGCAGATATTTTTTTTCATAACGCCAAA
50	AATGGATTATTTACACCTCGGCCTTGC
51	TTTTGACGCTAGTAATAGAATA
52	CGTACTATGGTTGCTACTTAGTAATAA
53	TTTTTAGCAATACTTCTTTGAGCAAATTAACCGTTGTTTTTT
54	CATCACCGCCTTGAAAAATCCATTTGAG
55	TAGAAGAACTGGTGAGGTAGAAGTATT
56	TGGTACCAGCAACAATTCGATATTTTTG
57	ACAAGAGAGCGAGGGTAGGCCGGAGAC
58	CGTGGCACAGACAACACCAGTCACAC
59	ATCACCTTGCCAGCAAAGCAACAGTGCCACGCTTTTTT
60	CGTTATTAAATAATATATATAACGTGCTCGCGCTACAGTGAG
61	TATTAAATCTTTACAAGAAGATAAA
62	GACCCAATCGTAGAACAATATTACCATTAAAAGCTA
63	CATTCTGAAATACCATTGCAACAGGAAAAATTTTT
64	TTTTTATAGCCCTAAAACATCGCCGCCAGCCTACA
65	ACAGACAAACTAGCGCTTAATGCGCCGTAACCACGAA
66	AATGATACCGAACGAACCAATATCCCTGA
67	TTAGTCTTTAAGCGTAAAAAGGGA
68	TTTTTGAGAGCCAGTGAACCTCTGTCCATCTTGACGAGCACGAAGC
69	GATTCGGTCAGTATTAACACTTGCCGGCG
70	AGACCTTTGCCCCACACCCGTTGGCAGATTCAACTCG
71	ATTCGCCTGATTGCTTACACGTAAAAC
72	GAGACTTAGATACTAAAGGAATTAATTTCAACCGCT
73	TTTTTTACAGTAACGAGTCAACAACAACCATAGAAAGG
74	AATCCTGATTTCAACAGAAATC

75	GCAATCATCATCAACCTAAAACGTCTTAAACGCGGA
76	ATTTTAAAGGCAGTTTCAGCGGAGTGAGAATCGGCTT
77	TTTTTCTTCTGAATAATGGAAGGGCAACGGCTGAGGAAGT
78	CCATATCAAAAACAGCATGGTAAAATACGTA
79	AGAACGTCACCTTTTGCTAAACAACTGAATTTTTAC
80	AACATAAGACGCTGAGAAAGTACCTTAACGTCAGATGAATATTTTT
81	AACGTAACATTTTTCATACAGAGGCTTTGAGGTTTTT
82	GCACATTCCTGATTTGAATACCAAGTCTGTATGGCTACGAAG
83	AAAATCGTAGAACAATAAATTGCGTAG
84	TAGAGAGTATGAGAAAGCTATTTTTG
85	CTCCAAAATCACAAATTAATGC
86	TTTTTCAAAGCGAACCAGACCGTTTTAATTCGAGCTTTTTTT
87	TTTGCGGATGCTGAAAAAATGGTCAATAACCTGTTTAGCTATATTTTT
88	TTTGCGGGATATAAAGAACGG
89	GCAGGGAGTTCAGGTTTTTACATCGG
90	TTTTTTATATTCGGTCGCTGAGCCCACGCATAACCGATTTTT
91	ACGAGGGTAGTTAGAACATAT
92	TTTTTACTAAAGACAAGCTGCGGTCAGTTGGCAAAGTTTGGATTATATTTTT
93	TTCCAATTACCCTTGAAAGGAATTTATTCATCACTA
94	ATGCCAGACTCAGCAGCGAAAGTTATTTGTCAGATGATG
95	TTTCCAGACGTTTTTTTTTTAGTAGCGAATAA
96	GATATAAAGAGGTTTTTTTTCAAAAGAATACA
97	ATAAGAATAAACACCAAAACGCGAGAA
98	ATAAAGTTTAGTGGGCTT
99	AATGGAAACAGTACACCACTATATGTA
100	TTTTTTAGGTTGGGTTATATATTTTTAACCTCCGGCTTTTTT
101	AATGGTTCAGCATAAGTCCTTCATTACC
102	CAATCGCAAGACGACGCCAATAGCAA
103	AACTAAGGTAAATAGAAGGCCATATTTA
104	AATTGAGAATCGCTTACTAGAAAAAG
105	GAAAAATAATACAATAGTAATGCAGAACGCGCCTTTTT
106	TTTTAGCGTAGGAAGATGTGAGTGAATAACTTACCTTAAATC
107	GGTATTCTAAATCAGATAGTAATTCTG
108	CCTTAAGGCGATATATTTTAGTTAGAGAATACGCC
109	TTATAAATACCCTTCTGACCTAAATTTTTTTT
110	TTTTTTAATGCTGTAGCTCAAGCTTAATTGCTGAATATTTTT
111	CTGGAAGTGGTTGCTCCTTTTGATTACTAATAAGG
112	TTTTTATTTTCGAGCCAGTAATAAATTTCATGACCGTGTG
113	TCCAGACAAAGCAATTTCATTTGAAAAAATTAAGGCG

114	ACAATAAAGTACCGACAATTTTCAATTAA
115	AACATGTAATAACAGTAATCATATGCG
116	TTTTTTGTTTATCAATCCCATGAGAGACTATAAATCAATATAACAA
117	GCGCACAATAAACAACATCTGATGCTTTT
118	GCAAGAACGCGATTACATTTGGAATCATAATTATCC
119	GAGAATTAACACATACAAATGCC
120	TTTTTCAGGGAAGCTATAAAAACAGTGCCCG
121	CCAACGCTAATACAATTATGTACCGTAACTTACCCTCAACTC
122	GCCTCCGTCGAAGGCTGAGACTCCTAACAGTTTAAA
123	TTTTTCAAAATAAACAGCCATATTGTACCGCCCCTCAGAG
124	TCCAAATAAGTCACCGTTTTT
125	GTTTAACGTGTATAGCCTGAAACATG
126	CCCTGCCTTATTAAGGAGGGTTGAT
127	TATACAAGAGACAGGCGGATAAGTGTTACAGAACGG
128	TCTTAAACAGACCGCCACACCCTCAGAACCGCCTTTTT
129	ACCCTTATCCTGATAATTGAGCGCTAACCTATTACAATAGGA
130	GGTGGCAACAGCATTAGGAGAATAACATAAAAATTTTT
131	ATAACAAAAATCCTGAACAAAGTCAGAGGGATTTTTT
132	TTTTTGGGTTTTGCTCAGTACAGGATTAGGATTAGCGTTTTT
133	AGGAGGTTTAATTTATCCTTTCCAGAGCCTATCTTTCCAATT
134	AAAGATTTCGGAATATCAGAGAAATTGAACACGAAAATAGCA
135	TTTTTACCCTCAGACTTGATATCAATAATCGGCTGATTTGCCAGTTATTTTT
136	CCACCCGAGGTGTTATCATTCCAAGCCGAGCGTCCAA
137	CAGGGATAGCAAGCCTTCCGGAATAGGTGTAAAACGATCTTA
138	AGCGAGAGGCTTTTTTTTTTTGCTGAATCCC
139	TTGCTACTGAGTTTTTTTTTTTCGTCACCAGT
140	AATCACGATAGCAGCGCGTTTTCATCTTGCCATTCAG
141	AAGTTACCAGAAGGACAGCCAAAGACA
142	ACCATTAGCAAGTAAGGTAAATATTGACCTCAGAACCCCT
143	GGTGGCCTCCCCTTTTCATAATCAAAGACTGTAGCA
144	TTTTTCATATGGTTTACCAGCCAATCAATAGAAAATTTTTTT
145	AAAGACCAGAAAGGTTGAGGC
146	ACCGATTGAGGCCACCATTGGCC
147	AGTCTCTGAATGACAGGCCACCACCAGAGCCGCTTTTT
148	AGCCAGACAGACGACCCTCAGAGCCGCCGGCGACAAACA
149	ATCCAGCCCTTTTGCCGGAAACGTCTCGGTCATAACAAATAA
150	AACCATCCAGTAGATTCATTAAA
151	CCGTAATCAGGAATTAGACCGTCACCGACTTGATTTTT
152	AGCCGCCACCGGAAATTCACCATT

153	TTTTTCAGAGCCACCACCGGAACCAATTATCAGCCAGCAA
154	CAGAGGAGGGAAGAAAAGTAAGCAGTTACCGATCATTAA
155	TTTTTCGCCAGCATTTTACCGTTATTTTGTAACCGAGGAAACGCGC
156	AGGTATGGAAAGCAATAATAACATCATAGCCGTTCA
157	TTGATATTCACAAGCCCGCCACCCT
158	TATTAGCGTGGCATTTACCAATGA
159	GGCGATTAAGTTGAAGCTTGCATGCTTTTT
160	CGGATTCTCCGTGGGAGCTAACTCACAT
161	ACATGGCTTTTAACGGGGTCAG
162	CTTCTGACCTGAAATGCGCGAACTGTTTTT
163	CCGAGTAAAAGAGTCAAATATCAAACCC
164	AAAGCCAACGCTCTTAGGCAGAGGCTTTTT
165	AAAATCATAGGTCTCCTAATTTACGAGC
166	TGCCTTTAGCGTCAATCACCGGAACTTTTT
167	ACCACGGAATAAGTTTCCAGTAAGCGTC
168	CTTGCATCAGTGAGGCAATCAATATCTTCATTCAGTGAATAAGG
169	AATGATAGTGAATTTAGTAGAAACCAACCGATAGTTGCGCCGAC
Structure staples	
1	ACAGTC GTCAATCATATGTACCCTAAC
2	GACTTC CGATGAACGGTAATCGTAAAACTAAAAA
3	ATCAGCTCATTTTTCGGTTGATAATCAG GGTTAT
4	GATCAG CAATAGGAACGCCATCAGCAT GTGCTA
5	TCAGAG TAATTCGCGTCTGGCCTTCC ATACGA
6	CGGTAGCAGGCGAAAATCCTG TCGTTG
7	CGGCATGGTGGTTCCGAAATCGGCAA TCTTCG
8	CCAGCTGCATTAATGAAT CGTTGA
9	CTACTC CGCTGGTTTGCCCCTTGCGTATTGGGCG
10	GGTCAT TTTGCAACGCGCGGGGAGAGG
11	ATCAAACACTATCATAACCCTC CTCACA
12	GTATCA GTTTAAACGAGAATGACCATAA
13	TTCGGC CCTCAAATGCTTTAAACAGTT
14	CAGAACCAGACGACGATAAAAACCAAAAT GTGAGT
15	GTCGAT GAGCAAAATCAGGTCTTTACCCTGACTAT
16	TCTCAT AGGAATTACGAGGCATAGTAA
17	TCATGG CGCTAGGGCGCTGGCAAAGCG
18	CGATGG AGGAAGGGAAGAAAGCGAAAGGAGCGAT
19	CTCGTTAGAATCAGGTGTAGCGGTCACG GCAAGT
20	GGAGCTAAACAGGAGGCCGGG ATCGGT
21	CGCTAA GATGTC TAAAGGATTTTAGACAGGA

22	ATAGACATTATCATTTGCGG GCGTAC
23	CTTTAAGAAACCACCAGAAGGAGCGG AGTCTG
24	GAAGGTTATCTAAAATAT GATGCT
25	GATTCG TAAAAGTTTGAGTAATTAGAGCCGTCAA
26	GCACAT AACAAGGAGCACTAACAACTA
27	TCTGGA AAACCGCATAGGCTGGCTGACC
28	ACAGATGAACGGTGTACAGAC CGTTCA
29	CAGGAAAGTACAACGGAGATTTGTATCAT AATGAC
30	AACTTA TTAGAAAAAAAAGGCTCCAAAA
31	TTTAGA CCCATCAAGAGTAATCTTGACAAGAACCG
32	CATTAG CTAAAACACTCATCTTTGACC
33	ATTCGG TGATTCGTCGCTATTAATTAA
34	GTAGCG AATTCCCTTAGAATCCTTGAAAACATAG
35	AGAGGCGAATTATTCATTTC GTCCAT
36	CTTGCTTCTGTAAAGAAACAAACATCAA TACTCT
37	TTTTACCTGAGCAAAAGAAGA TGTTGA
38	AAGCGAGGTTTTGAAGCCTTA ACGATC
39	CCGCAAGATTAGTTGCTATTTTGCAC GCTTCA
40	CGGGTATTAAACCAAGTA GGTCAC
41	TGTCGA CTCCCGACTTGCGGCGTTTTTATTTTCA
42	ATCGCT AATCACTCATCGAGAACAAGC
43	AACTTA TTAGAAAAAAAAGGCTCCAAAA
44	TAATTTTTTCACGTTGAAAAT GATAAG
45	CTCCCGTAACGATCTAAAGTTTTGTCGTC CAAGCA
46	TTGCTG ATTCCCTTTAATTGTATCGGTTTATCAGC
47	GGCATG ACAAACTACAACGCCTGTAGC
48	GGAGCACAGACAGCCCTCATAG ATCAGC
49	AGCACT AACATGGCATGATTAAGACTC
50	GACATC AGCCTTACGCAGTATGTTAGCAAACGTA
51	AACCCACAAGAATTGAGTTA TGACGT
52	ATACCCAAAAGAACATGAAATAGCAATA TAACTG
53	CTTACAATAATAAGAGCAAGA GAATCC

Table S5. Open-Set and Close-Set DNA for linear branch shape DNA nanocage transformation to square and pyramid shape.

Name	Sequence
Open DNA set (backbone structure)	
1	TGTGAGGAGGGTTATGATAGTGTTTGAT
2	TTATGGTCATTCTCGTTTAAACTGATAC
3	AACTGTTTAAAGCATTTGAGGGCCGAA
4	ACTCACATTTTGGTTTTTATCGTCGTCTGGTTCTG
5	ATAGTCAGGGTAAAGACCTGATTTTGCTCATCGAC
6	TTACTATGCCTCGTAATTCCTATGAGA
7	GGTCAGCCAGCCTATGCGGTTTTCCAGA
8	TGAACGGTCTGTACACCGTTCATCTGT
9	GTCATTATGATACAAATCTCCGTTGTACTTTCCTG
10	TTTTGGAGCCTTTTTTTTCTAATAAGTT
11	CGGTTCTTGTCAAGATTACTCTTGATGGGTCTAAA
12	GGTCAAAGATGAGTGTTTTAGCTAATG
13	TTTTGGAGCCTTTTTTTTCTAATAAGTT
14	CTTATCATTTTCAACGTGAAAAAATTA
15	TGCTTGGACGACAAAACTTTAGATCGTTACGGGAG
16	GCTGATAAACCGATACAATTAAAGGGAATCAGCAA
17	GCTACAGGCGTTGTAGTTTGTCATGCC
18	GCTGATCTATGAGGGCTGTCTGTGCTCC
Open DNA set (bevel structure)	
1	GTTAGGGTACATATGATTGACGACTGT
2	TTTTTAGTTTTACGATTACCGTTCATCGGAAGTC
3	ATAACCCTGATTATCAACCGAAAAATGAGCTGAT
4	TAGCACATGCTGATGGCGTTCCTATTGCTGATC
5	TCGTATGGAAGGCCAGACGCGAATTACTCTGA
6	CAACGACAGGATTTTCGCCTGCTACCG
7	CGAAGATTGCCGATTTCGGAACCACCATGCCG
8	TCAACGATTCATTAATGCAGCTGG
9	CGCCCAATACGCAAGGGGCAAACCAGCGGAGTAG
10	CCTCTCCCCGCGCGTTGCAAAATGACC
11	CGCTTTGCCAGCGCCCTAGCGCCATGA
12	ATCGCTCCTTTCGCTTTCTTCCCTTCCTCCATCG
13	ACT TGC CGT GAC CGC TAC ACC TGA TTC TAA CGA G
14	ACC GAT CCC GGC CTC CTG TTT AGC TCC
15	TCC TGT CTA AAA TCC CTT TAG ACA TCT TAG CG
16	GTA CGC CCG CAA AAT GAT AAT GTC TAT

17	CAG ACT CCG CTC CTT CTG GTG GTT TCT TAA AG
18	AGC ATC ATA TTT TAG ATA ACC TTC
19	TTG ACG GCT CTA ATT ACT CAA ACT TTT ACG AAT C
20	TAG TTG TTA GTG CTC CTT GTT ATG TGC
21	TTA ATT AAT AGC GAC GAA TCA CCG AAT
22	CTA TGT TTT CAA GGA TTC TAA GGG AAT TCG CTA C
23	ATG GAC GAA ATG AAT AAT TCG CCT CT
24	AGA GTA TTG ATG TTT GTT TCT TTA CAG AAG CAA G
25	TCA ACA TCT TCT TTT GCT CAG GTA AAA
26	GAT CGT TAA GGC TTC AAA ACC TCG CTT
27	TGA AGC GTG CAA AAT AGC AAC TAA TCT TGC GG
28	GTG ACC TAC TTG GTT TAA TAC CCG
29	TGA AAA TAA AAA CGC CGC AAG TCG GGA GTC GAC A
30	GCT TGT TCT CGA TGA GTG ATT AGC GAT
31	GAG TCT TAA TCA TGC CAT GTT AGT GCT
32	TAC GTT TGC TAA CAT ACT GCG TAA GGC TGA TGT C
33	ACG TCA TAA CTC AAT TCT TGT GGG TT
34	CAG TTA TAT TGC TAT TTC ATG TTC TTT TGG GTA T
35	GGA TTC TCT TGC TCT TAT TAT TGT AAG
Close DNA set (backbone structure)	
1	CCTCAAATGCTTTAAACAAATCAGGTCTTTACCCTGACTAT
2	AGGAATTACGAGGCATACCAGACGACGATAAAAACCAAAAT
3	CTAAAACACTCATCTTAAAGTACAACGGAGATTTGTATCAT
4	ACAGATGAACGGTGTACCAAGAGTAATCTTGACAAGAACCG
5	TAATTTTTTCACGTTGACTTTAATTGTATCGGTTTATCAGC
6	ACAAACTACAACGCCTCGTAACGATCTAAAGTTTTGTCGTC
Close DNA set (bevel structure)	
1	ATCAGCTCATTTTTTAACCAATAGCGCGTCTGGCCTTCC
2	CGATGAACGGTAATCATATGTACCCCGGTTGATAATCAG
3	CGCTGGTTTGCCCCAATGGTGGTTCCGAAATCGGCAA
4	CCAGCTGCATTAATGAATCGGCTTTGCGTATTGGGCG
5	AGGAAGGGAAGAAAGGCGCTGGCAAGTGTAGCGGTCACG
6	CTCGTTAGAATCAGAGCGGGAGCTGGATTTTAGACAGGA
7	TAAAAGTTTGAGTAAAAGAAACCACCAGAAGGAGCGG
8	GAAGGTTATCTAAAATATCTTTGATTAGAGCCGTCAA
9	CTTGCTTCTGTAAATCGTCGCTATTCCTTGAAAACATAG
10	AGAGGCGAATTATTCAGAAGATGATGAAACAAACATCAA
11	CTCCCGACTTGCGGGAAGATTAGTTGCTATTTTGCAC
12	CGGGTATTAAACCAAGTACCGCCCGTTTTTATTTTCA

13	AACCCACAAGAATTGGCAAGAAACAATGAAATAGCAATA
14	ATACCCAAAAGAACTGGCATGATTATGTTAGCAAACGTA
DNA connection set (for pyramid)	
1	TTTTTTCTGGTGCCAAGGGGGATGTGTTCAAGTT
2	TTTTTCGCTCATGGGCCAACAGAGATTCTGCAA
3	TTTTTAATGGTTTGACAAATTCTTACTTAGAACC
4	TTTTTGCCATTTGGTAGCGACAGAATTCAGTAT
5	TGCGAACACTGGTAATAAGTTTT
6	GATGATACAGGAGTGTGAGTAGATTT
7	ATAAGTTTTGATGATACAGGAGTGTGAGTAGA
8	TTTAGTTTGACCTTTCGCAGGTGGTACGAC
DNA connection set (for square)	
1	TGCGAACACTGGTAATAAGTTTT
2	GATGATACAGGAGTGTGAGTAGATTT
3	ATAAGTTTTGATGATACAGGAGTGTGAGTAGA
4	TTTAGTTTGACCTTTCGCAGGTGGTACGAC

DNA origami	Dimension	Designed size (nm)	2D size by AFM (nm)	Particle diameter by NTA (nm)
Rectangular	2D	~ 60 × 90	~ 60 × 100	~ 55 (major peak)
Triangular	2D	~ 120 for each side	~ 120 for each side	~ 66 (major peak)
Linear	1D/2D	~ 160 in length	~ 165 for the extended structure	~ 80 (major peak)
Square	3D	~ 40 × 40 × 40	~ 50 × 50	~ 57 (major peak)
Pyramid	3D	~ 40 × 40 × 29	~ 50 × 45	~ 41 (major peak)

Table S6. The size comparison for the designed dimension and the measurement by AFM and NTA.
